# The proto-oncoprotein FBI-1 interacts with MBD3 to recruit the Mi-2/NuRD-HDAC complex and BCoR and to silence *p21WAF/CDKN1A* by DNA methylation

**DOI:** 10.1093/nar/gkt359

**Published:** 2013-05-08

**Authors:** Won-Il Choi, Bu-Nam Jeon, Jae-Hyeon Yoon, Dong-In Koh, Myung-Hwa Kim, Mi-Young Yu, Kyung-Mi Lee, Youngsoo Kim, Kyunggon Kim, Sujin Susanne Hur, Choong-Eun Lee, Kyung-Sup Kim, Man-Wook Hur

**Affiliations:** ^1^Department of Biochemistry and Molecular Biology, BK21 Project for Medical Science, Severance Biomedical Research Institute, Yonsei University School of Medicine, 50 Yonsei-Ro, SeoDaeMoon-Gu, Seoul, 120-752, Korea, ^2^Department of Biomedical Sciences & Biomedical Engineering, Seoul National University College of Medicine, 103 Daehangno, Seoul 110-799, Korea, ^3^Sookmyung Girls’ High School, 91 Dogok-Dong, Gangnam-Gu, Seoul, 135-505, Korea and ^4^Department of Biological Science, Sungkyunkwan University, Suwon 440-746, Korea

## Abstract

The tumour-suppressor gene *CDKN1A* (encoding p21Waf/Cip1) is thought to be epigenetically repressed in cancer cells. FBI-1 (ZBTB7A) is a proto-oncogenic transcription factor repressing the alternative reading frame and *p21WAF/CDKN1A* genes of the p53 pathway. FBI-1 interacts directly with MBD3 (methyl-CpG–binding domain protein 3) in the nucleus. We demonstrated that FBI-1 binds both non-methylated and methylated DNA and that MBD3 is recruited to the *CDKN1A* promoter through its interaction with FBI-1, where it enhances transcriptional repression by FBI-1. FBI-1 also interacts with the co-repressors nuclear receptor corepressor (NCoR), silencing mediator for retinoid and thyroid receptors (SMRT) and BCL-6 corepressor (BCoR) to repress transcription. MBD3 regulates a molecular interaction between the co-repressor and FBI-1. MBD3 decreases the interaction between FBI-1 and NCoR/SMRT but increases the interaction between FBI-1 and BCoR. Because MBD3 is a subunit of the Mi-2 autoantigen (Mi-2)/nucleosome remodelling and histone deacetylase (NuRD)-HDAC complex, FBI-1 recruits the Mi-2/NuRD-HDAC complex via MBD3. BCoR interacts with the Mi-2/NuRD-HDAC complex, DNMTs and HP1. MBD3 and BCoR play a significant role in the recruitment of the Mi-2/NuRD-HDAC complex– and the NuRD complex–associated proteins, DNMTs and HP. By recruiting DNMTs and HP1, Mi-2/NuRD-HDAC complex appears to play key roles in epigenetic repression of *CDKN1A* by DNA methylation.

## INTRODUCTION

Factor that binds to the inducer of short transcripts of human immunodeficiency virus-1 (FBI-1) (ZBTB7A) is a recently characterized proto-oncoprotein of the POZ-domain Krüppel-like (POK) family of transcription factors. It plays important roles in the cell cycle, cell differentiation, proliferation, fatty acid synthesis, immune responses and oncogenesis. FBI-1 promotes cellular transformation by repressing alternative reading frame (ARF), p21 and Rb expression, and has been shown to promote cell proliferation and oncogenesis in the thymus, liver and spleen in transgenic mice (1–3). We have demonstrated that expression of the fatty acid synthase (FASN), which is important in palmitate synthesis and cell proliferation in cancer cells, is potently activated by FBI-1 in the presence of sterol regulatory element binding protein-1 (SREBP-1) (4). FBI-1 has also been shown to enhance NF-κB mediated transcription by an interaction between the POZ-domain and the Rel homology domain of NF-κB (5). The mouse counterpart of FBI-1, the leukaemia/lymphoma-related factor, is co-immunoprecipitated and co-localized with proto-oncoprotein Bcl-6 (6). FBI-1 is expressed in almost all tissues. Serial analysis of gene expression (SAGE), oncomine data and immunohistochemistry analysis have shown that the expression of FBI-1 is increased in various cancer tissues.

DNA methylation is one of the epigenetic events that can regulate gene expression [(7) and references therein] and is important in transcriptional repression, genomic imprinting, X-chromosome inactivation and genomic stability. DNA from mammalian tissues is methylated at 70% of all CpG sites (8). Key exceptions to this global methylation are the CpG islands, which are frequently located in the 5′-regulatory and/or promoter region. CpG islands are non-methylated in germ cells, in early embryos and in all somatic tissues (9). For the majority of genes, the CpG islands of their 5′-promoter regions are not methylated and they are expressed.

DNA methylation is catalysed by DNA (cytosine-5)-methyltransferase enzymes (DNMT 1, 3a or 3b) (10). Aberrant DNA methylation patterns have been associated with a large number of human malignancies and are found in two distinct forms: hypermethylation and hypomethylation when compared with normal tissue [(11,12) and references therein]. Hypermethylation, which typically occurs at CpG islands, represses transcription at the promoter regions of tumour-suppressor genes, including p16INK4a, p53, RB1 and BRCA1 [(12,13) and references therein]. Global hypomethylation has also been implicated in the development and progression of cancer through genome instability (14).

The methyl-CpG–binding domain proteins (MBDs) read and bind methylated DNA. MBD proteins recruit additional chromatin remodelling proteins that can modify histones to form compact silent chromatin. Accordingly, they are mediators of epigenetic transcriptional silencing of the hypermethylated promoters, as was first demonstrated for methyl CpG binding protein 2 (MeCP2) (15). The mammalian MBD protein class contains five members, MBD1, MBD2, MBD3, MBD4 and MeCP2 (16). MBD3 is unique in that it cannot bind to methylated DNA. With the exception of MBD4, which is involved in DNA repair, all MBD proteins (MBD1, MBD2 and MeCP2) associate with histone deacetylases (HDACs) and couple DNA methylation to transcriptional silencing through the modification of chromatin [(17) and references therein]. The molecular interaction between the transcription repression domain of the MBD proteins and the co-repressor complexes is important for the transcriptional repression of methylated promoters [(18) and references therein].

MBD3 is a component of the Mi-2 autoantigen (Mi-2)/nucleosome remodelling and histone deacetylase (NuRD)-HDAC complex (Mi-2/nuclesome remodelling and deacetylase), a chromatin-remodelling complex that contains a nucleosome remodelling ATPase, HDAC1 and HDAC2, and metastasis-associated protein 2 (MTA2) [(19) and references therein]. MBD3 has no intrinsic DNA-binding activity and is targeted to the methylated promoter by interacting with MBD2 and maintains transcriptionally repressed chromatin [(18) and references therein; (20)]. Interestingly, MBD3 protein was shown to be associated with the proximal promoter of *CDKN1A* and was released on treatment of cancer cells with a HDAC inhibitor (21). The function and mechanism of MBD3 association at the promoter remains uncharacterized. By recruiting HDACs and DNA methyltransferases (DNMTs), MBD3 acts as a transcriptional repressor, which is important in oncogenic transformation and cell proliferation (22). SAGE analysis shows that FBI-1 and MBD3 are expressed ubiquitously in most human tissues, with increased expression in various cancer tissues.

DNMT1 is the most abundant DNMT in mammalian cells and considered to be the key maintenance methyltransferase in mammals (23). DNMT1 predominantly methylates hemimethylated CpG dinucleotides in the mammalian genome. The enzyme is 7–100-fold more active on hemimethylated versus unmethylated DNA substrates *in vitro*, but it is still more active with respect to *de novo* methylation than other DNMTs. DNMT3a and DNMT3b are *de novo* methyltransferases and can mediate methylation-independent gene repression. DNMT3a can co-localize with the heterochromatin protein (HP1) and the methyl-CpG–binding protein, and can also interact with DNMT1. DNMTs can be targeted to endogenous genes by interaction with site-specific transcriptional repressor proteins, such as PML-RAR, which cause hypermethylation of target genes in cancer cells (24). It was proposed that DNMT1 represses the p21 promoter by the mechanism that is not methylation dependent (25,26). However, it was shown by chromatin immunoprecipitation (ChIP) assays that Myc is required for efficient recruitment of DNMT3a to the *CDKN1A* promoter and the DNMT3a is required for gene silencing by promoter DNA methylation (27).

The cyclin-dependent kinase inhibitor (*CDKN1A,* p21) is a major player in mammalian cell cycle progression [(28) and references therein]. It inhibits cyclin/cdk2 complexes and interrupts cell cycle progression*. CDKN1A* is a transcriptional target of p53 and plays a crucial role in mediating growth arrest when cells are exposed to DNA-damaging agents, suggesting that p21 has a tumour suppression role (29). Overexpression of p21 results in G_1_-, G_2_- or S-phase arrest. A variety of other factors, including Sp1/Sp3, Smads, Ap2, STAT, BRCA1, E2F-1/E2F-3 and C/EBPα and ß, activate the transcription of *CDKN1A*. In addition to its role in the DNA-damage response, p21 has also been implicated in terminal differentiation, replicative senescence and protection from p53-dependent and -independent apoptosis [(30) and references therein]. Recently, we have shown that FBI-1 represses the transcription of *CDKN1A* by binding competitively with p53 and Sp1 at the distal and proximal regulatory elements, respectively, which results in deacetylation of histones near the proximal promoter (1).

Epigenetic silencing of *CDKN1A* gene expression in cancer cells by histone modification and promoter DNA methylation is suggested by the upregulation of *CDKN1A* gene expression after treatment with the HDAC inhibitor trichostatin A (TSA) or the DNA methylation inhibitor 5-aza-2'-deoxycytidine (31). The *CDKN1A* promoter was also shown to be bound by MBD3 and was released on treatment with the HDAC inhibitor, TSA (21). These data suggest that the *CDKN1A* promoter is methylated and that histone deacetylation, DNA methylation and the binding of MBD3 proteins may be related.

In this study, we investigated the function and mechanism of the interaction between FBI-1 and MBD3 in the epigenetic transcriptional regulation of *CDKN1A,* which involves modification of histones and DNA methylation at the proximal *CDKN1A* promoter. This study illuminates new roles of the proto-oncoproteins FBI-1, MBD3 and BCL-6 corepressor (BCoR) in the epigenetic regulation of p21 expression, and perhaps in oncogenesis.

## MATERIALS AND METHODS

### Plasmid construction

Human MBD3 (GenBank: BC009372) cDNA was amplified from a cDNA library using oligonucleotide polymerase chain reaction (PCR) primers: forward, 5′-GATCCTCGAGACCATGGAGCGGAAGAGCCCG-3′; reverse, 5′-GATCAAGCTTGACGTGCTCCATCTCCGG-3′. The amplified cDNA fragment was inserted into the XhoI and HindII site of pcDNA3.1 (Invitrogen, Carlsbad, CA). All plasmid constructs were verified by sequencing. The *CDKN1A*-Luc plasmid was kindly provided by Dr. Yoshihiro Sowa of Kyoto Perpetual University of Medicine (Kyoto, Japan). Plasmids, including pPac-PL-FBI-1, pGL2-CDKN1A-Luc (−133 bp), pGL2-5x(FRE)-Luc and pCMX-GAL4-ZF-FBI-1, co-repressors and pCMX-VP16-co-repressors were reported elsewhere (2). The pPac-PL-MBD3 plasmid was prepared by cloning the cDNA fragment into pPac-PL. All plasmid constructs were verified by sequencing. To prepare the glutathione S-transferase (GST)-POZFBI-1 and GST-ZFFBI-1 fusion protein expression plasmids, cDNA fragments encoding the POZ-domain and ZF of FBI-1 were subcloned into pGEX4T3 (Amersham Biosciences, NJ) and reported elsewhere (1). Antibodies against GAPDH, GST, MBD3 and Flag-Tag were purchased from Chemicon (Temecula, CA), Calbiochem (San Diego, CA), SantaCruz Biotechnology (Santa Cruz, CA) and Sigma (St. Louis, MO), respectively.

### *In vitro* methylation of pG5-5x(FRE)-Luc reporter plasmid and preparation of FRE and methylated FRE

pG5-5x(FRE)-Luc reporter plasmid was incubated with SssI (New England Biolabs, Beverly, MA) to methylate the plasmid according to the manufacturer’s instruction. Methylation was confirmed by restriction digestion with BstUI and HpaII for 16 h at 60°C and agarose-gel electrophoresis. Oligonucleotide sequences used for the FRE (or methylated FRE) and FBI-1 interactions are as follows: FRE (forward, 5′-GATCCGAGCGCGGGTCCCGCCTC-3′; reverse, 5′-GATCGAGGCGGGACCCGCGCTCG-3′), methylated oligonucleotide probes were synthesized by Intergrated DNA Technologies (Coralville, IA). Methylated oligonucleotide probes used are as follows: Me-FRE (forward, 5′-GATC/5-Methyl-dC/GAG/5-Methyl-dC/G/5-Methyl-dC/GGGTCC/5-Methyl-dC/GCCTC-3′; reverse, 5′-GATCGAGG/5-Methyl-dC/GGGACC/5-Methyl-dC/G/5-Methyl-dC/GCT/5-Methyl-dC/G-3′).

### Chromatin immunoprecipitation (ChIP)/reChIP assays

The molecular interaction between FBI-1 and MBD3 on the pGL2-5x(FRE)-Luc was analysed by ChIP assays. HEK293 cells were transfected with pGL2-5x(FRE)-Luc or methylated pGL2-5x(FRE)-Luc (2 μg), pcDNA3.0-FLAG-FBI-1 and pcDNA3.1-MBD3 (2 μg) using Lipofectamine Plus. Cells were fixed with formaldehyde (final 1%) to cross-link FBI-1 and MBD3 on the 5x(FRE). The remainder of the ChIP procedure is reported elsewhere (2). PCR reactions of chromatin-immunoprecipitated DNA were carried out using oligonucleotide primer sets designed to amplify the region flanking the FRE elements of *CDKN1A*: forward, 5′-GGTACTGTAACTGAGCTA-3′; reverse, 5′-GATCCAGATCTCGAGCTA-3′.

For ChIP-reChIP assays of FBI-1 and MBD3 binding at the CDKN1A proximal promoter, the first ChIP samples using anti-FBI-1 antibody or anti-MBD3 antibody were diluted 10 times with dilution buffer (15 mM Tris–HCl, pH 8.1, 1% Triton X-100, 1 mM EDTA, 150 mM NaCl) and immunoprecipitated using anti-MBD3 or anti-FBI-1 antibodies. Negative control ChIP assays were carried out with rabbit IgG antibody. The 3′-UTR of *CDKN1A* was used as a negative control region of protein–DNA interaction using the following oligonucleotide primers: forward, 5′-TCCTTCCCATCGCTGTCACA-3′; reverse, 5′-GTCACCCTGCCCAACCTTAG-3′.

The histone modifications were investigated by ChIP assay. HEK293 cells were transfected with pcDNA3.0-FLAG-FBI-1 and/or pcDNA3.1-MBD3 (2 μg) using Lipofectamine Plus (Invitrogen, Carlsbad, CA). Also, primary human dermal fibroblast neonatal (HDFn) cells were transfected with various combinations of pcDNA3.0-FLAG-FBI-1 (5 μg), pcDNA3.1-MBD3 (5 μg), FBI-1 siRNA (100 pmoles) and/or MBD3 siRNA (100 pmoles) using the Neon Kit of the Neon Transfection System (Invitrogen, Carlsbad, CA) according to the manufacturer’s instructions (Supplementary Table 1). After 48 h, cells were harvested and the ChIP assay was performed as described above. Rabbit polyclonal IgG (Labfrontier, Seoul, Korea), anti-acetyl-Histone 3, anti-acetyl-Histone 4, anti-trimethyl-Histone H3 lysine 4 (H3K4-Me3) and anti-trimethyl-Histone H3 lysine 9 (H3K9-Me3) (Upstate, Lake Placid, NY) antibodies were used. To analyse modifications of histone 3 and histone 4 around the proximal *CDKN1A* promoter, the following oligonucleotide primers were used: forward, 5′-GCGCTGGGCAGCCAGGAGCC-3′; reverse, 5′-CGCTCTCTCACCTCCTCT-3′.

To analyse the DNA methylation at the proximal *CDKN1A* promoter by ChIP, a Me-DIP kit (Diagenode, Liège, Belgium) designed to immunoprecipitate methylated DNA with an anti-methylated-DNA antibody was used.

### Immunoprecipitation assays

Cells were washed with PBS, pelleted by centrifugation and resuspended in lysis buffer (20 mM Tris–HCl, pH 7.5, 150 mM NaCl, 10% glycerol and 1% Triton X-100) supplemented with a protease inhibitor cocktail. Cell lysate was collected by centrifugation, pre-cleared by incubation with protein A-Sepharose Fast Flow (Sigma, St. Louis, MO), pre-equilibrated with lysis buffer on a rotating platform, centrifuged and the supernatant was collected. Supernatants were incubated with the primary antibody and further incubated with protein G-Sepharose Fast Flow beads pre-equilibrated in lysis buffer. Beads collected by centrifugation were washed and resuspended in an equal volume of 5 × SDS loading buffer. Immunoprecipitated proteins were separated by 12% sodium dodecyl sulphate-polyacrylamide gel electrophoresis (SDS-PAGE). Western blot assays were performed as described above.

### DNA methylation analysis by bisulfite DNA sequencing

Genomic DNA was purified with the Wizard genomic DNA purification kit (Promega, Madison, WI). Methylation analyses were performed by bisulfite conversion of genomic DNA using the EpiXploreTM Methyl Detection kit (Clontech, Palo Alto, CA). The primer sequences used to amplify the *CDKN1A* promoter region were sense 5′-AGGAGGGAAGTGTTTTTTTGTAGTA-3′ and antisense 5′-ACAACTACTCACACCTCAACTAAC-3′. The PCR product was cloned using the pGEM®-T Easy vector System I kit (Promega, Madison, WI). Mini-scale plasmid DNA was prepared from >30 individual transformed *Escherichia coli* and were sequenced.

## RESULTS

### Proto-oncoprotein FBI-1 interacts with MBD3

To understand the biological functions of the proto-oncogene FBI-1, we isolated a nuclear protein complex containing FBI-1 from stable HEK293-TREx-FLAG-FBI-1 cells by immunoprecipitation using anti-FLAG-M2-agarose beads. The immunoprecipitates were separated by a 2D isoelectric focusing gel (Supplementary Figure S1A and B). A 34 kDa protein band was excised from the gel, digested with trypsin and subjected to matrix-assisted laser desorption/ionization–time of flight mass spectrometry analysis. Database searches for peptide mass fingerprinting using the MASCOT program (Matrix Science Ltd., London, UK) revealed that the protein was MBD3 (Supplementary Figure S2A and B).

**Figure 1.**
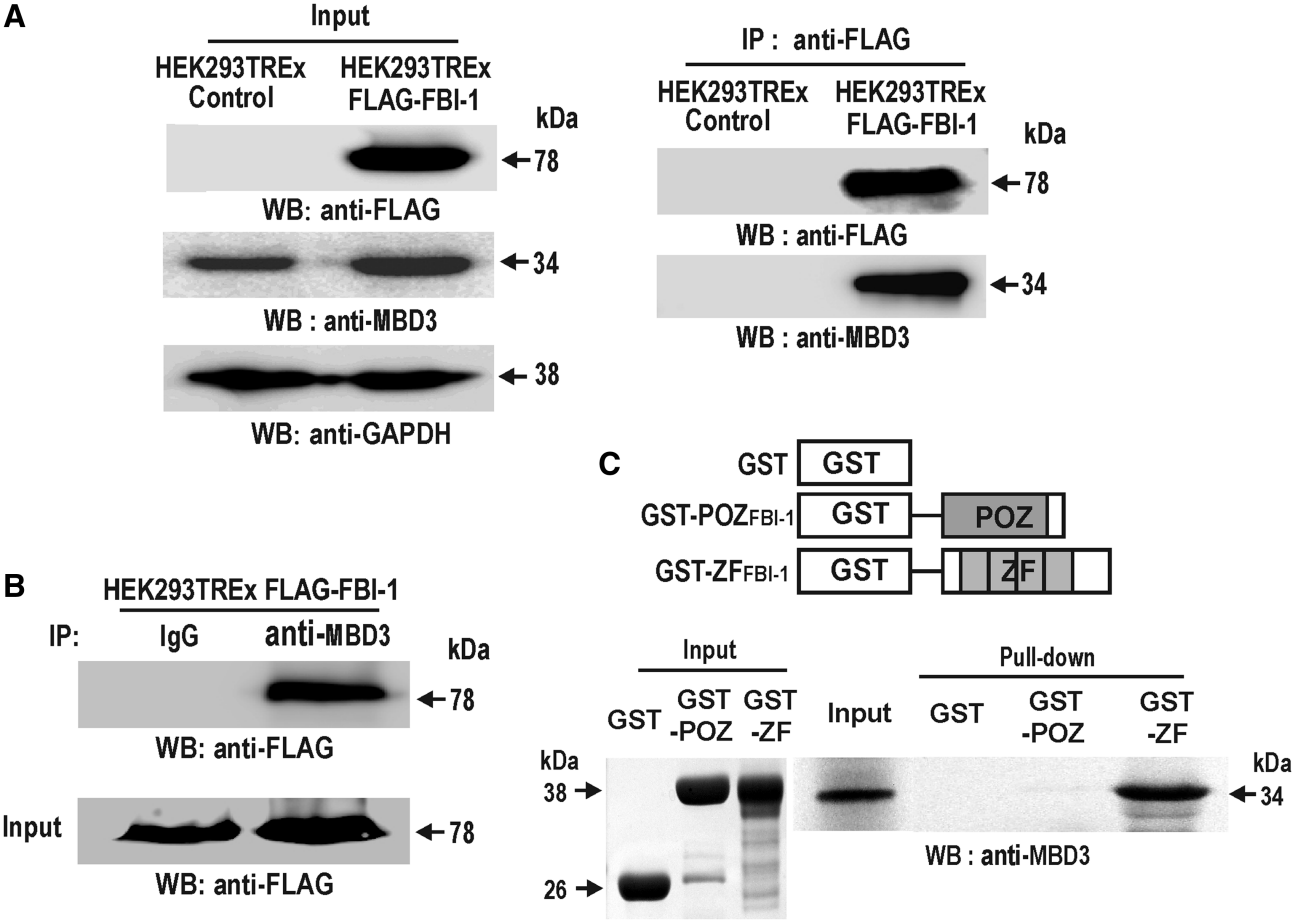
FBI-1 interacts directly with MBD3. (**A**) Co-IP and western blot analysis. HEK293T-REX-control and stable HEK293T-REX-FLAG-FBI-1 cell nuclear extracts were immunoprecipitated with anti-FLAG M2 agarose beads, and the precipitates were analysed by western blot using the indicated antibodies. (**B**) Reverse co-IP of (**A**). The same nuclear extracts were immunoprecipitated with an antibody against MBD3, and the precipitates were analysed by western blot using the anti-FLAG antibody. (**C**) GST-fusion protein pull-down assays. Schematic diagram of the domains of FBI-1 tested. Recombinant GST protein, GST-POZFBI-1 and GST-ZFDBDFBI-1 were incubated with *in vitro* synthesized [^35^S]-methionine-labelled MBD3, precipitated, resolved by 10% SDS-PAGE and analysed by autoradiography.

To characterize the interaction between MBD3 and FBI-1, nuclear extracts prepared from the control and doxycycline-induced stable HEK293-TREx-FLAG-FBI-1 cells were co-immunoprecipitated using the anti-FLAG M2 antibody or IgG-conjugated agarose beads as a control. Western blotting analysis of the immunoprecipitates using antibodies against MBD3 and FLAG showed that FBI-1 interacts with MBD3; reciprocal co-immunoprecipitations (co-IPs) of the same nuclear extract and western blot assays also demonstrated specific binding between endogenous MBD3 and FBI-1 (Figure 1A and B). GST-fusion protein pull-down assays showed that MBD3 interacted directly with the zinc-finger DNA-binding domain of FBI-1 (GST-ZF; Figure 1C). FBI-1 co-localized with the MBD3 protein in the nucleus of HeLa cells transfected with expression vectors of FLAG-FBI-1 and/or His-tagged MBD3 (data not shown). The above data, along with proteomic data, suggest that FBI-1 directly interacts with MBD3 in nucleus.

### MBD3 enhances transcriptional repression of *CDKN1A* by FBI-1

**Figure 2.**
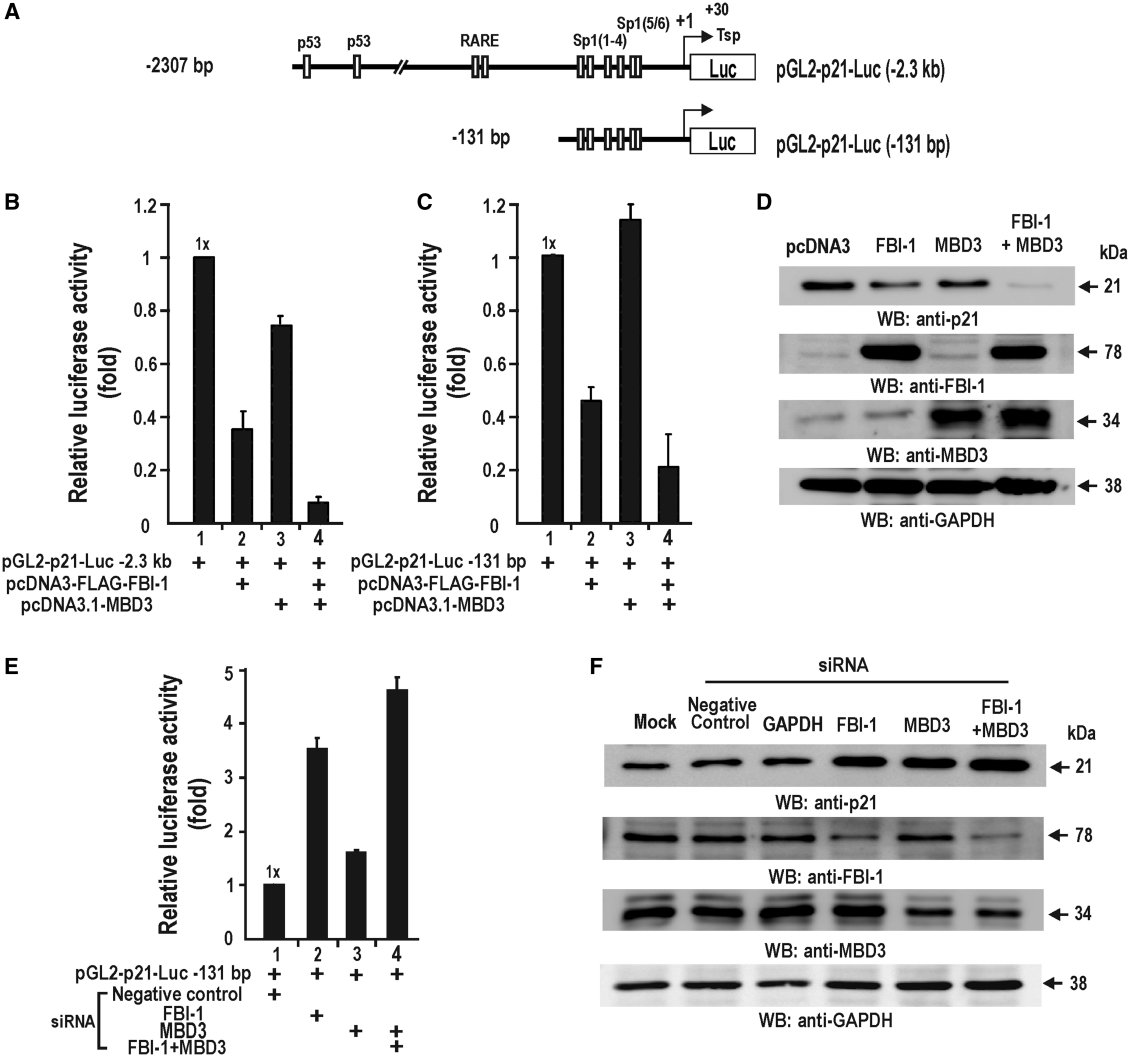
MBD3 enhances transcriptional repression of the *CDKN1A* gene by FBI-1. (**A**) Structures of the two *CDKN1A-*luc fusion reporter constructs tested. (**B** and **C**) Transient transcription assays in HEK293 cells. Cells were transfected with each luciferase reporter construct and the expression vectors for MBD3 and FBI-1. The average of three independent assays is shown; bars represent standard deviations. (**D**) Western blot analysis. HEK293 cells were transfected with MBD3 and/or FBI-1 expression vector. Cell lysates were analysed for endogenous p21 expression. Ectopic FBI-1 and MBD3 expression potently repress endogenous *CDKN1A* expression. (**E**) Transient transcription assays in HEK293 cells. Knock-down of FBI-1 or/and MBD3 derepressed reporter gene expression from the *p21WAF/CDKN1A*-Luc (−131 bp) construct. Luciferase activities were normalized to protein concentration. The average of three independent experiments is shown. Bars represent standard deviations. (**F**) Western blot analysis. HEK293 cells were transfected with MBD3 siRNA and/or FBI-1 siRNA. Cell lysates were analysed for endogenous p21 expression. Knock-down of endogenous FBI-1 and/or MBD3 expression increases endogenous p21 expression. Negative control, scrambled siRNA.

Using the two promoter-luciferase reporter fusion constructs that contained either distal and/or proximal FBI-1 target sites (1) (Figure 2A), we examined whether MBD3 affected transcription repression of *CDKN1A* by FBI-1 in HEK293 cells. As we reported previously, FBI-1 repressed transcription of the reporter constructs, and MBD3 alone weakly repressed transcription, possibly by interacting with endogenous FBI-1 (1). However, when FBI-1 and MBD3 were co-transfected, strong transcriptional repression was observed for both promoter constructs (Figure 2B and C), suggesting that the FBI-1-MBD3 interaction is important for transcriptional repression on the proximal promoter. Western blot assays of cell lysates prepared from the HeLa cells transfected with combinations of FBI-1 and/or MBD3 expression vectors showed that MBD3-FBI-1 co-expression also potently repressed expression of endogenous p21 (Figure 2D).

Using a loss of function approach, we tested whether endogenous FBI-1 and MBD3 are important for the transcriptional repression of *CDKN1A* in HEK293 cells. Knock-down of FBI-1 resulted in derepression of the *CDKN1A* gene as reported previously (1). Knock-down of MBD3 also resulted in derepression. Knock-down of both FBI-1 and MBD3 resulted in the most robust derepression (Figure 2E and Supplementary Figure S3). Western blot assays of the cell lysates prepared from the cells transfected with siRNAs against FBI-1 and/or MBD3 revealed an increased expression of endogenous p21 (Figure 2F). These data suggest that MBD3 can enhance the transcriptional repression of *CDKN1A* by FBI-1 and that the FBI-1-MBD interaction may be significant in cell cycle regulation by repressing p21 expression.

### FBI-1 binds to the methylated and non-methylated FBI-1–binding DNA element (FRE) of the *CDKN1A* promoter *in vitro*. MBD3 binding is increased by FBI-1 and enhances transcriptional repression by FBI-1

**Figure 3.**
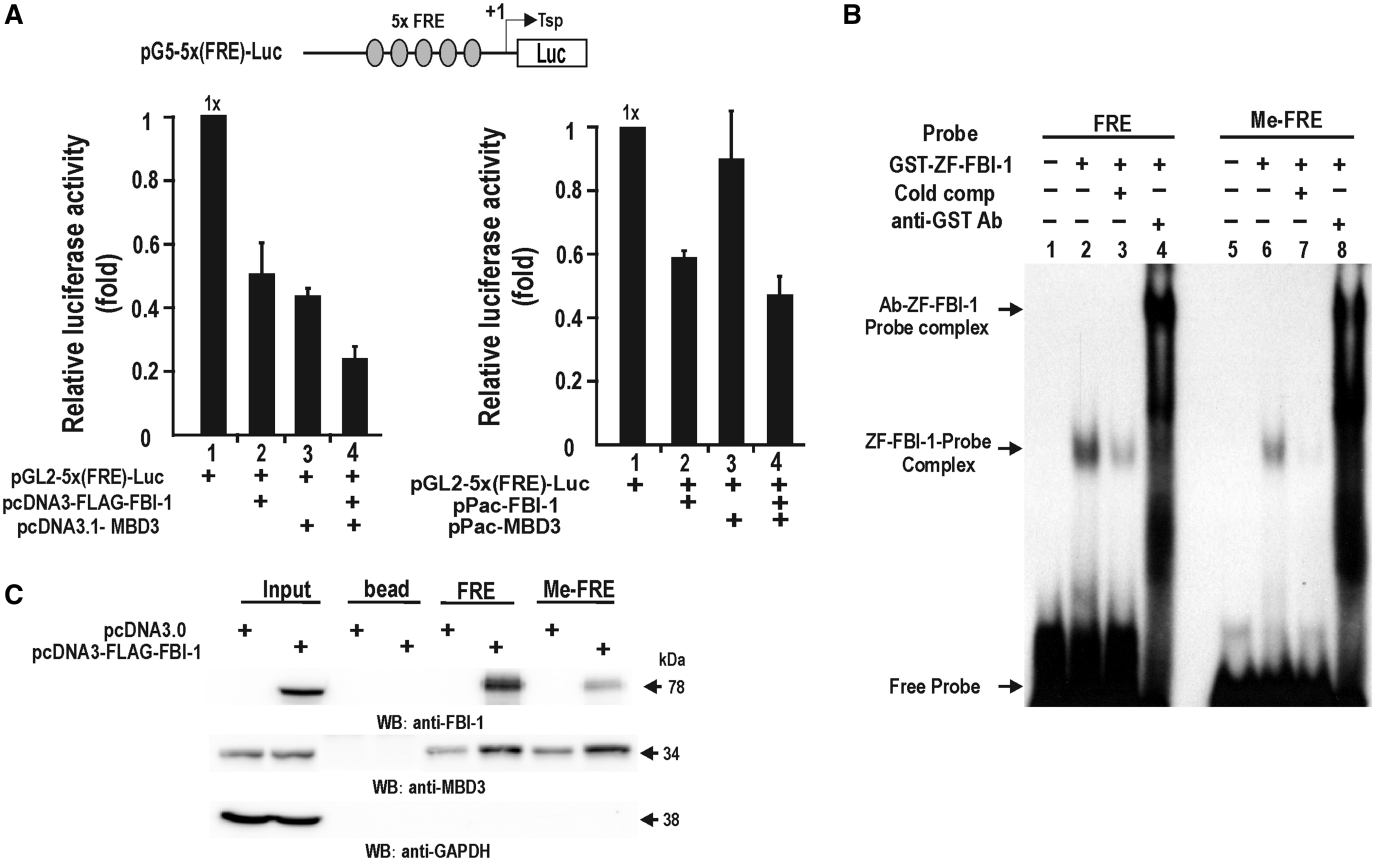
MBD3 enhances transcriptional repression of pG5-5x(FRE)-Luc by FBI-1. FBI-1 binds to both methylated and unmethylated FRE elements of *CDKN1A*, and MBD3 binding to the FRE probes is increased by ectopic FBI-1. (**A**) Transient transcription assays in HEK293 cells and Drosophila SL2 cells. Cells were transfected with the reporter construct, mammalian or Drosophila (pPac) expression vector of FBI-1 and/or MBD3. All data shown are the average of three independent assays; bars represent standard deviations. (**B**) EMSA. ^32^P-labelled FRE and methylated FRE probes were incubated with recombinant GST-ZF-FBI-1 protein (500 ng), separated by 4% non-denaturing polyacrylamide gel electrophoresis, and the dried gel was exposed to radiographic film. (**C**) Oligonucleotide pull-down assay. Nuclear extracts from HEK293 control or HEK293 cells transfected with the FLAG-FBI-1 expression vector were incubated with biotinylated double stranded oligonucleotides corresponding to the FRE and methylated FRE. The precipitated complexes were analysed by western blotting using the anti-FLAG and anti-MBD3 antibodies. GAPDH was used as a control.

MBD3 enhances transcriptional repression by FBI-1 bound to the FRE (GC-box #3, −104 to −90 bp) in the proximal promoter of *CDKN1A*. We tested whether transcriptional repression of pG5-5x(FRE)-Luc by FBI-1 could be further repressed by MBD3 in HEK293 cells and Drosophila SL2 cells lacking mammalian MBD3 and FBI-1. FBI-1 repressed transcription in both cell lines; MBD3 could not repress transcription of pG5-5x(FRE)-Luc in SL2 cells because SL2 cells lack endogenous human FBI-1. However, co-expression of MBD3 and FBI-1 in SL2 cells resulted in more significant repression (Figure 3A). These data suggested that transcriptional repression by MBD3 requires FBI-1 bound to the FRE.

MBD proteins (MBD1, 2, 4, 5 and MeCP2) can bind to methylated DNA. Although MBD3 cannot bind to DNA directly, it may be targeted to the FRE by interacting with FBI-1 bound at the proximal promoter. By electro-mobility shift assay (EMSA), we demonstrated that the recombinant zinc-finger DNA-binding domain of FBI-1 (ZF-FBI-1) bound to the FRE probe and, interestingly, that FBI-1 also bound to the methylated FRE probe, although rather weakly. This binding was eliminated by excess unlabelled probe (Figure 3B). These data suggest that FBI-1, like KAISO, may be involved in recognizing both methylated and non-methylated DNA elements, such as FRE, and silencing of FBI-1 target genes.

We also tested whether FBI-1 and MBD3 could bind the probes using DNA pull-down assays. The cell lysates prepared from HEK293 cells transfected with pcDNA3 or pcDNA3-FLAG-FBI-1 expression vector were incubated with FRE or methylated FRE probe conjugated to streptavidin-agarose beads. Western blot analysis of the precipitates with anti-FBI and anti-MBD3 antibodies showed that FBI-1 can bind both non-methylated FRE and methylated FRE probes and that ectopic FBI-1 enhances binding of MBD3 to the probes (Figure 3C).

### FBI-1 binds to both methylated and non-methylated FRE in HEK293 and SL2 cells, and recruits MBD3 to the *CDKN1A* promoter

**Figure 4.**
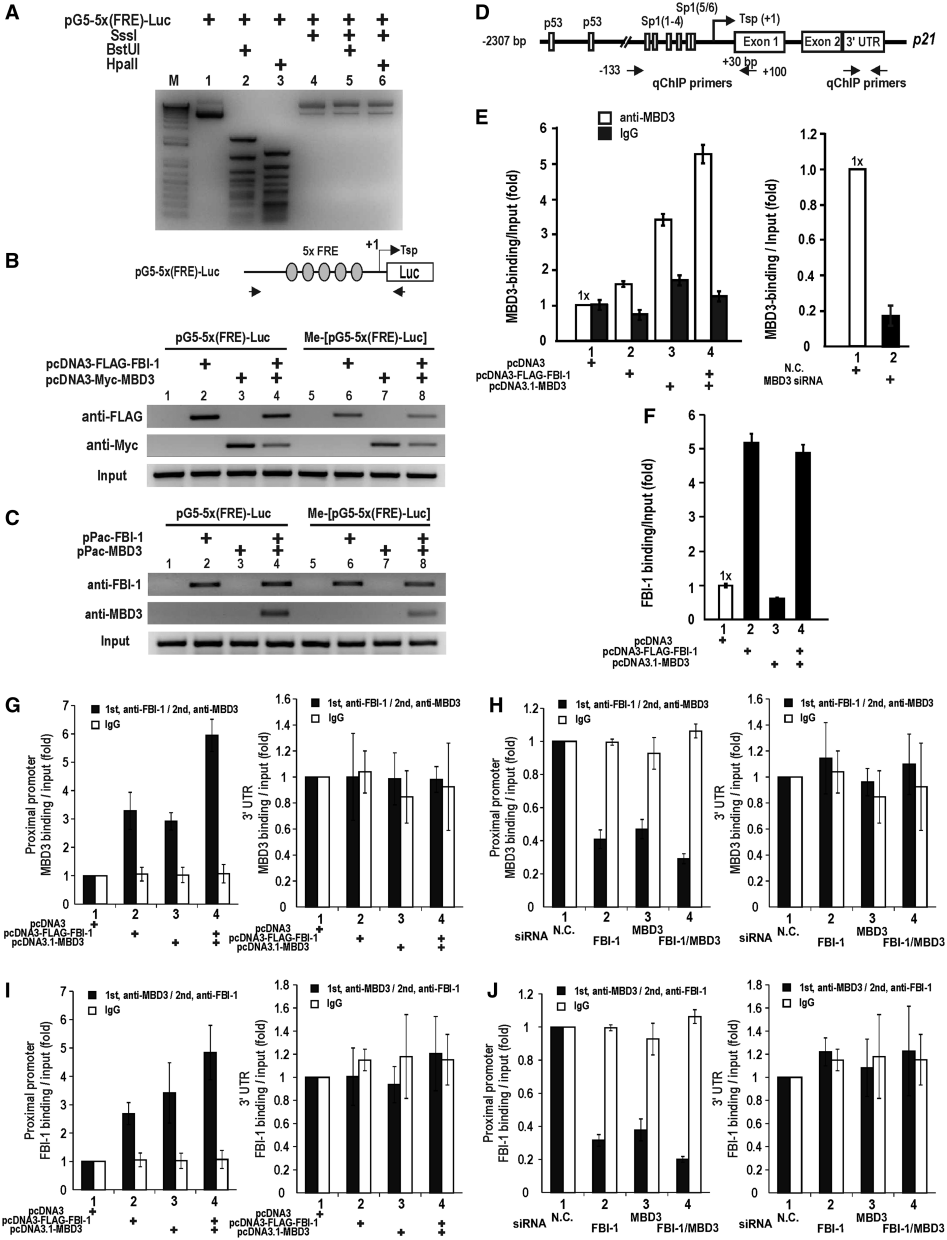
MBD3 binding to the FRE element requires FBI-1 *in vivo*. (**A**) Methylation of the FBI-1 responsive reporter, pG5-5x(FRE)-Luc, with the methylase SssI. Plasmid methylation was tested by digestion with methylated DNA-specific endonucleases BstUI and HpaII. M, DNA size marker. Lanes 1–3, unmethylated plasmid. Lanes 4–6, methylated plasmid. (**B**) ChIP assays of FBI-1 and MBD3 binding on the proximal promoter of pG5-5x(FRE)-Luc and the methylated plasmids transfected into HEK293 cells. ChIP primer sets were designed to amplify the region flanking the proximal FRE elements. Arrows indicate the primer-binding sites. (**C**) ChIP assays of FBI-1 and MBD3 binding in Drosophila SL2 cells lacking mammalian transcription factors. MBD3 binding requires FBI-1. (**D**) Structure of the human *CDKN1A* gene promoter. Arrows at the promoter regions indicate the locations of ChIP PCR primer-binding sites. (**E**) qChIP assays of MBD3 binding to the endogenous *CDKN1A* promoter under ectopic expression of FBI-1 and/or MBD3, or knock-down of MBD3 in HEK293 cells. MBD3 binding is increased by ectopic FBI-1 and/or MBD3 (**F**) qChIP assays of FBI-1 in the presence of ectopic FBI-1 and/or MBD3. (**G–J**) ChIP-reChIP assays of FBI-1 and MBD3 binding at the proximal *CDKN1A* promoter by ectopic expression or knock-down of either FBI-1 or MBD3. The negative controls were IgG and the 3′-UTR.

Using ChIP assays, we also investigated whether FBI-1 and MBD3 could bind to the proximal *CDKN1A* promoter that contains six copies of the methylated and non-methylated FRE of pG5-(5xFRE)-Luc *in vivo* (Figure 4A). The pG5-(5xFRE)-Luc plasmid and the methylated pG5-(5xFRE)-Luc plasmid used in the assays were prepared by treatment with the CpG methyltransferase, SssI. Methylation of the plasmid with SssI resulted in complete resistance to digestion by the methylated DNA-specific restriction enzymes, BstUI and HpaII (Figure 4A). We transfected HEK293 and Drosophila SL2 cells with either the pG5-5x(FRE)-Luc plasmid or the methylated pG5-5x(FRE)-Luc plasmid and analysed FBI-1 and MBD3 binding by ChIP assay. As in the DNA pull-down assays, FBI-1 was able to bind both non-methylated and methylated FRE in both types of cells. MBD3 bound the FRE alone in HEK293 cells, most likely by interacting with endogenous FBI-1. MBD3 bound to the FRE only in the presence of co-transfected FBI-1 in SL2 cells (Figure 4B and C lane 3). These data suggest that MBD3 binds to the promoter by interacting with endogenous FBI-1.

We examined if ectopic expression or knock-down of MBD3 expression affected MBD3 binding at the endogenous *CDKN1A* promoter using qChIP assays. FBI-1 expression increased endogenous MBD3 binding, as did MBD3 overexpression; co-expression of FBI-1 and MBD3 further increased MBD3 binding (Figure 4D and E left). Knock-down of MBD3 expression nearly eliminated MBD3 binding in the region (Figure 4D and E and Supplementary Figure S3). In contrast, ectopic MBD3 did not affect FBI-1 binding (Figure 4F).

Furthermore, we examined whether ectopic or endogenous (as demonstrated by knock-down) FBI-1 and MBD3 could be bound to the proximal promoter of *p21WAF/CDKN1A* simultaneously via ChIP-reChIP assays with anti-FBI-1 and anti-MBD3 antibodies. The results showed that FBI-1 and MBD3 were bound to each other. In contrast, neither FBI-1 nor MBD3 was bound to the 3′-UTR region of the gene used as a negative control (Figure 4G–J). These data suggested that MBD3 is recruited to the non-methylated FRE and methylated FRE *in vivo* by molecular interaction with FBI-1.

### MBD3 recruited by FBI-1 affects the molecular interaction between FBI-1 and co-repressors, and drives FBI-1 to interact with BCoR.

Previously, we showed that FBI-1 can interact with co-represssor–HDAC complexes such as nuclear receptor corepressor (NCoR), silencing mediator for retinoid and thyroid receptors (SMRT) and BCoR and that the FBI-1–co-repressor complex deacetylates histones H3 and H4 to repress transcription (1). Because MBD3 enhances transcriptional repression by FBI-1, we investigated whether MBD3 affects the protein interactions between co-repressors and FBI-1.

**Figure 5.**
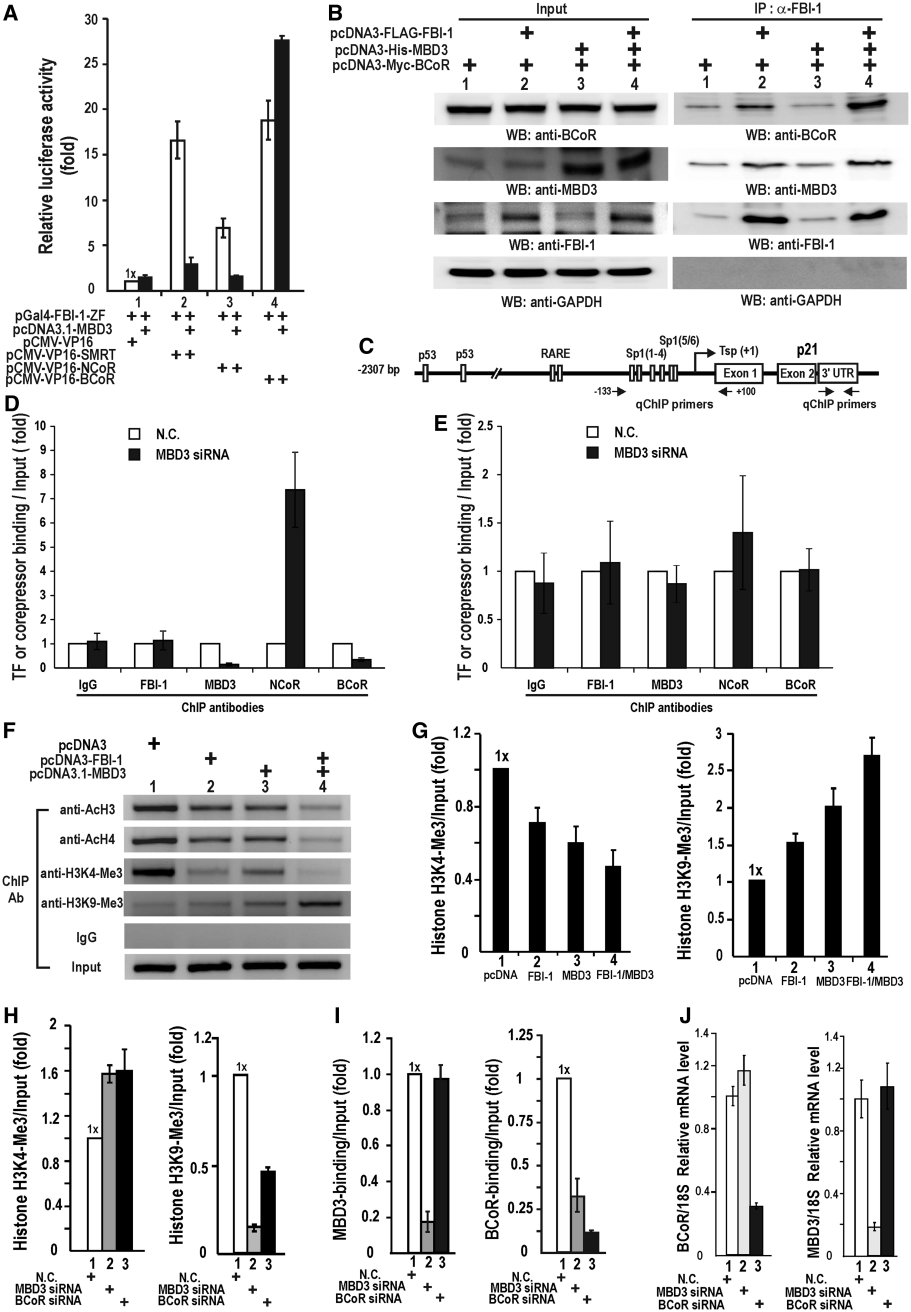
MBD3 increases the protein interaction between FBI-1 and co-repressor BCoR. The MBD3–FBI-1 interaction and BCoR recruitment decreases histone acetylation and histone H3K4-Me3 but increases histone H3K9-Me3. (**A**) Mammalian two-hybrid assay of molecular interaction between co-repressor and FBI-1. HEK293 cells were transiently transfected with the plasmids indicated (200 ng of reporter plasmid, GAL4 fused FBI-1-ZF, VP-16 fused proteins and MBD3 expression vector). All data represent the average of three independent assays and bars represent standard deviations. (**B**) Co-IP and western blotting. HEK293 cells were transfected with the expression vectors, cell lysates were immunoprecipitated and analysed by western blotting using the indicated antibodies. (**C**) Structure of the human *CDKN1A* gene promoter. Arrows indicate the locations of ChIP PCR primer-binding sites. (**D**) qChIP assays of co-repressor switching (NCoR, BCoR) at the proximal *CDKN1A* promoter by knock-down of MBD3 expression. The binding of FBI-1 and MBD3 is also shown. The negative control was IgG. (**E**) qChIP assays of co-repressor, FBI-1 and MBD3 binding at the 3′-UTR of *CDKN1A*, a qChIP assay control DNA region. The negative control was IgG. (**F**) Semi-quantitative ChIP assay of histone modifications at the proximal promoter of endogenous *CDKN1A* gene by ectopic FBI-1 and/or MBD3 expression. Histone marks indicating active transcription (acetylated H3 and H4, H3K4-Me3) and repressed transcription (H3K9-Me3) were tested. (**G**) qChIP analysis of histone modifications (H3K4-Me3; H3K9-Me3) at the proximal promoter of the endogenous *CDKN1A* gene by ectopic expression or knock-down of MBD3 and/or MBD3 expression. N.C., negative control scrambled siRNA. (**H**) qChIP analysis of histone modifications (H3K4-Me3; H3K9-Me3) on the endogenous *CDKN1A* gene by knock-down of MBD3 or BCoR. N.C., negative control scrambled siRNA. (**I**). qChIP analysis of BCoR and MBD3 binding to the endogenous *CDKN1A* gene after knock-down of MBD3 or BCoR expression. (**J**) RT-qPCR analyses of knock-down efficiency of MBD3 or BCoR mRNA by siRNA.

We have shown that the POZ-domain (very weak, data not shown) and the zinc-fingers of FBI-1 interact with co-repressors (Figure 1D). Mammalian two-hybrid assays showed that the zinc-finger domain binding domain (ZFDBD) of FBI-1 interacts with SMRT, NCoR and BCoR and that its interaction with SMRT and BCoR were relatively strong compared with its interaction with NCoR. However, in the presence of MBD3, the interaction between FBI-1 and SMRT or NCoR is considerably decreased, but the interaction between BCoR and FBI-1 is increased (Figure 5A). Additionally, co-IP and western blot analysis revealed that BCoR, MBD3 and FBI-1 form a complex and that the molecular interaction with BCoR is increased by the presence of MBD3 (Figure 5B lane 4), while the molecular interaction between FBI-1 and MBD3 is proportional to the amount of FBI-1 present (Figure 5B). Due to the nature of mammalian two-hybrid assays, one might argue that the change in the strength of interactions might reflect the ability of the MBD3-associated NuRD complex to counteract the action of VP16 rather than provide a real indication of how well the proteins interact. However, the ChIP assays also showed that knock-down of MBD3 mRNA increased NCoR recruitment but decreased BCoR recruitment (Figure 5D and E). This suggests that by interacting with FBI-1, MBD3 decreases NCoR binding and increases BCoR binding, which is in line with the mammalian two-hybrid assay and co-IP results (Figure 5A and B).

Because interaction with the transcription co-repressor complex changes the modifications of chromatin, we investigated the changes in epigenetic histone marks by FBI-1, MBD3 and BCoR recruited to the proximal promoter of *CDKN1A*. As expected, given the transcriptional repressor roles of MBD3 and FBI-1, the two proteins decreased the acetylation of histones H3 and H4 and the trimethylation of histone H3K4. Interestingly, when co-expressed with FBI-1, MBD3 increased the trimethylation of histone H3K9, an indicator of transcriptionally repressed chromatin (Figure 5F and G).

We also examined whether a loss of function of MBD3 or BCoR affected the histone methylation marks of histone H3. Knock-down of either MBD3 or BCoR expression resulted in an increase in H3K4-Me3 and a decrease in H3K9-Me3, indicating transcription derepression (Figure 5H). While knock-down of MBD3 mRNA resulted in a decrease in BCoR binding, an absence of BCoR or a decrease in BCoR levels did not affect the recruitment of MBD3 to the proximal promoter, which indicates that BCoR recruitment requires MBD3 (Figure 5I and Supplementary Figure S9). Knock-down of MBD3 mRNA did not affect BCoR mRNA expression or vice versa (Figure 5J)

These data suggest that both MBD3 and BCoR have effects on the modification of histone H3 that are related to transcriptional repression. It appears that, once recruited to the target promoter by FBI-1, MBD3 causes a switch in the FBI-1 from interacting with co-repressor to BCoR, which results in the deacetylation of histones, demethylation of H3K4-Me3 and trimethylation of H3K9.

### MBD3 and FBI-1 cause DNA methylation of the *CDKN1A* proximal promoter by recruiting the Mi-2/NuRD-HDAC complex and BCoR

The *CDKN1A* gene is regulated by histone deacetylation (1) and potentially by DNA methylation of the proximal promoter, which is GC rich. Histone modification to H3K9-Me3 by MBD3 suggests that *CDKN1A* may be repressed by DNA methylation (Figure 5F–H).

**Figure 6.**
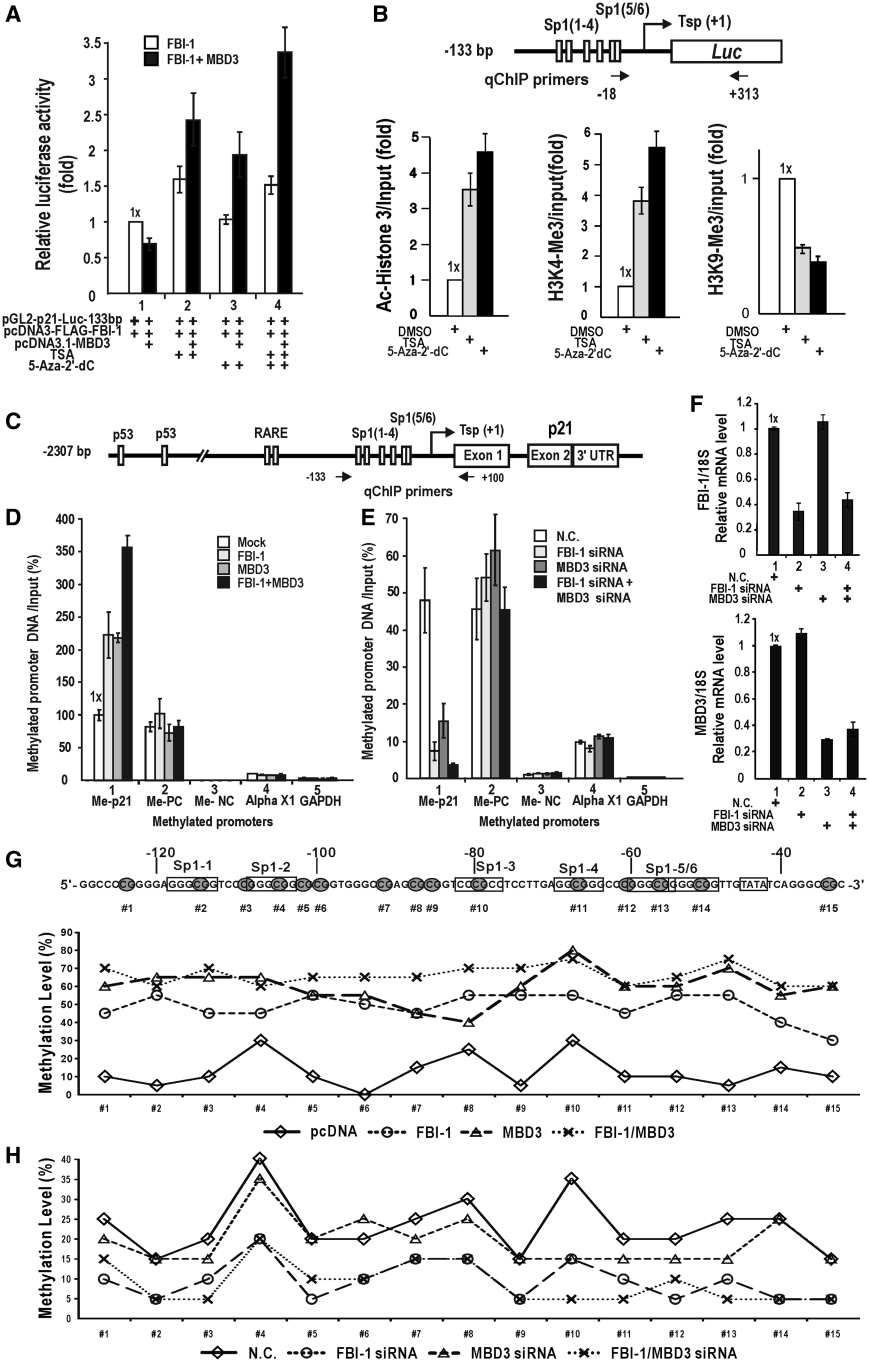
The *CDKN1A* gene is repressed by HDAC and DNA methylation; FBI-1 and MBD3 are important in DNA methylation. (**A**) Effect of the HDAC inhibitor, TSA (200 nM), and DNA methylation inhibitor, 5-aza-2′-deoxycytidine (1 µM), on the transcription of pGL2-*CDKN1A*-Luc (−131 bp) repressed by FBI-1 and/or MBD3. (**B**) qChIP assays of acetylated H3 and H4, H3K4-Me3 and H3K9-Me3 around the exogenous proximal *CDKN1A* promoter introduced by transfection, before and after treatment with TSA and 5-aza-2′-deoxy-cytidine. (**C**) Structure of the human *CDKN1A* gene promoter. Arrows at the promoter region indicate the locations of ChIP PCR primer sets. (**D–F**) Me-DIP assay of DNA methylation at the proximal promoter of the endogenous *CDKN1A* gene after ectopic expression or knock-down of FBI-1 and/or MBD3. Me-PC, methylation positive control (a positive signal is obtained for methylation) (Diagenode); Me-NC, methylation negative control (no signal is obtained for 0% methylation); GAPDH, GAPDH promoter (no signal is expected as this region is not methylated); Alpha X1, X-linked alpha-satellites (a positive signal is expected as it is a methylated region). (F) qRT-PCR showed that FBI-1 and MBD3 expression are knocked down efficiently. (**G** and **H**) Bisulfite DNA sequencing assays. The percentage of methylated cytosines in the *CDKN1A* promoter under various assay conditions is shown. Cells were transfected with expression vectors or siRNA containing knocked down FBI-1 and/or MBD3, and DNA was prepared for bisulfite sequencing. Individual PCR products were cloned, and 20 clones were sequenced. Filled circles on the proximal promoter sequence indicate the CpG sites that can be methylated.

Because a nucleosome-like structure was reported to be formed on the transfected promoter-reporter fusion plasmid DNA (32,33), we investigated whether the nucleosomal histone and the DNA around the minimal promoter fused to the luciferase gene were epigenetically modified. We compared reporter expression of the transfected cells treated with an inhibitor of HDACs (TSA) and/or DNMTs (5-aza-2′-dC) with that of the transfected cells treated with the control vehicle (DMSO). TSA and 5-aza-2′-dC derepressed transcription of the pGL2-CDKN1A-Luc (−131 bp) plasmid in HEK293 cells. In the presence of ectopic FBI-1 and MBD3, which increase BCoR binding, strong derepression was observed in the treatment with a combination of TSA and 5-aza-2′-dC (Figure 6A and Supplementary Figure S6). Furthermore, ChIP assays of the histone marks of transcriptional activation (H3K4-Me3) and repression (H3K9-Me3) around the region juxtaposed to the proximal promoter showed that acetylated histone H3 and H3K4-Me3 are increased and H3K9-Me3 is decreased by TSA or 5-aza-2′-dC treatment (Figure 6B). The data suggest that a nucleosome-like structure can be formed around the short proximal promoter of pGL2-*CDKN1A*-Luc (−133 bp) exogenously introduced and the *CDKN1A* promoter can be transcriptionally repressed by histone deacetylation and DNA methylation.

Accordingly, we examined whether the endogenous *CDKN1A* promoter of HEK293 cells could be methylated by FBI-1 and/or MBD3 using a Me-DIP assay kit (Methylated DNA Immunoprecipitation kit; Diagenode), which uses an antibody against methylated DNA. MBD3 or FBI-1 each increased methylation levels by >2-fold. In combination, they increased methylation by >3.5-fold; the two factors had no effect on the methylation status of positive or negative control promoters (Figure 6C and D). Knock-down of MBD3 and/or FBI-1 mRNA (Figure 6C and E) caused a significant decrease in DNA methylation of the *CDKN1A* proximal promoter and had no significant effect on the control promoters (Figure 6E and Supplementary Figure S8). The Me-DIP assays suggested that transcription of the *CDKN1A* proximal promoter might be repressed as a result of DNA methylation by the complex containing FBI-1 and MBD3. Furthermore, bisulfate DNA sequencing of the *CDKN1A* promoter showed that the core CpG bases of Sp1 binding GC-boxes #2, #3 and #4 were more heavily methylated than the Sp1 binding CpG bases of GC-boxes #1, #5 and #6. The methylation of CpG bases was significantly increased by expression of FBI-1 and/or MBD3 4.7 ± 0.09-fold (control 13–60%) for FBI-1 only, 3.8 ± 0.07-fold (control to 13–60%) for MBD3 only, and 5.2 ± 0.05-fold (control to 13–60%) for FBI-1 and MBD3 (Figure 6G and Supplementary Figure S7). Knock-down of FBI-1 and/or MBD3 mRNA significantly decreased CpG methylation 0.5 ± 0.05-fold (control 18–10%) for FBI-1 siRNA only, 1.1 ± 0.06-fold (control 18–10%) for MBD3 siRNA only and 0.5 ± 0.05-fold (control 18–10%) for FBI-1 and MBD3 siRNA (Figure 6H and Supplementary Figure S7). It appears that knock-down of ether FBI-1 or MBD3 weakly affects the methylation of the *CDKN1A* promoter. The knock-down experiments aim to decrease the FBI-1 and/or MBD3 already expressed at low levels that have little effect on methylation. These data imply that transcription of the endogenous *CDKN1A* promoter can be repressed by DNA methylation established by a series of molecular events including histone deacetylation, histone demethylation/methylation, which is initiated by binding of FBI-1 and MBD3.

### FBI-1, MBD3 and BCoR play roles in recruiting the Mi-2/NuRD-HDAC complex, DNMTs and HP1 to methylate *CDKN1A* promoter DNA.

**Figure 7.**
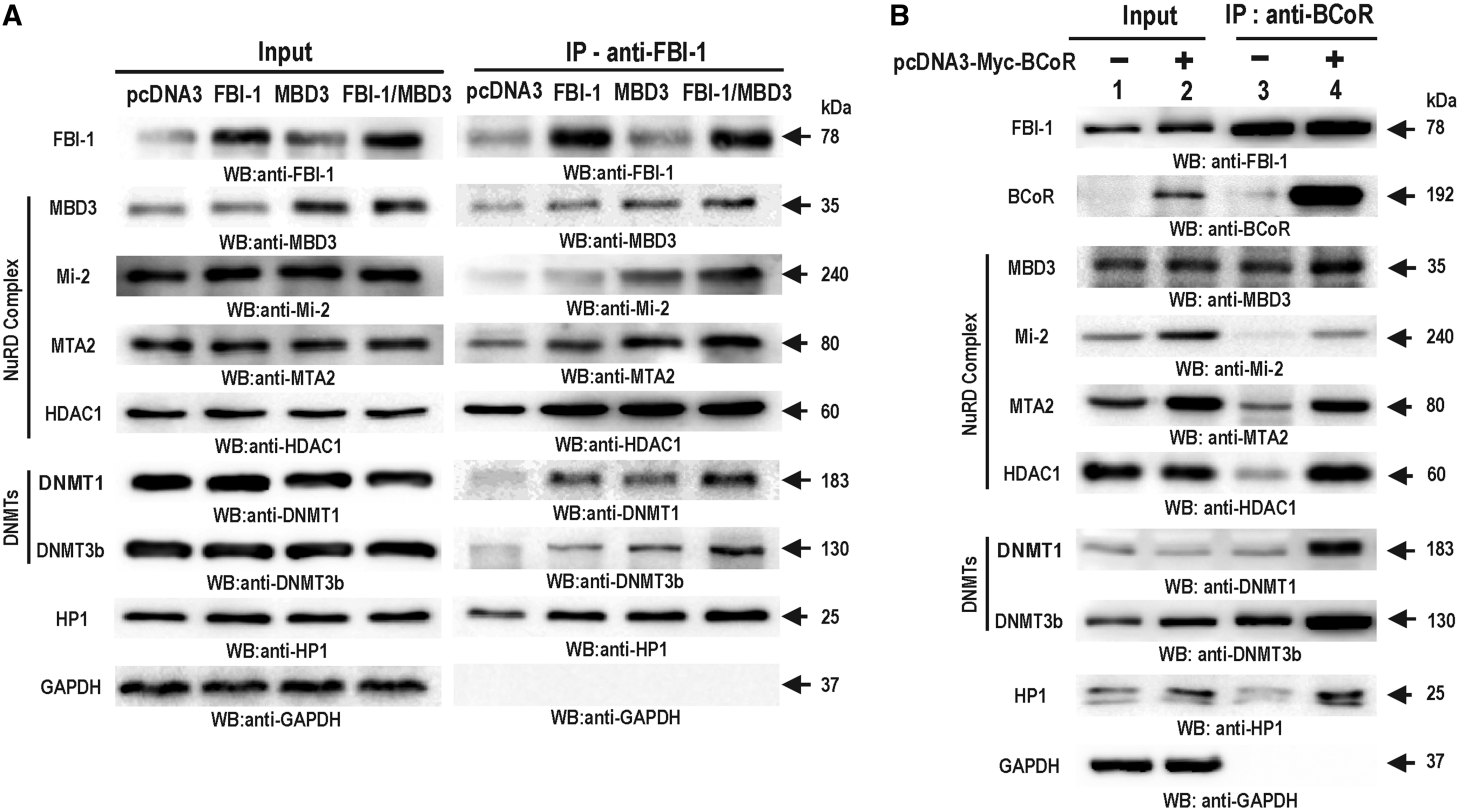
FBI-1 and BCoR interact with the MBD3-Mi-2/NuRD-HDAC complex, DNMT1, DNMT3b and HP1. (**A**) Co-IP and western blot analysis of FBI-1, MBD3, the Mi-2/NuRD-HDAC complex, DNMTs and HP1. HEK293 cells were transfected with the FBI-1 and/or MBD3 expression vector and immunoprecipitated using an anti-FBI-1 antibody. The immunoprecipitates were analysed for the presence of FBI-1, MBD3, the Mi-2/NuRD-HDAC complex, DNMTs and HP1. GAPDH was used as the loading control. (**B**) Co-IP and western blot analysis of FBI-1, BCoR, MBD3, the Mi-2/NuRD-HDAC complex, DNMTs and HP1. HEK293 cells were transfected with the BCoR expression vector and immunoprecipitated using an anti-BCoR antibody. The immunoprecipitates were analysed for the presence of FBI-1, MBD3, the Mi-2/NuRD-HDAC complex, DNMTs and HP1. GAPDH was used as the loading control.

Although MBD3 has no direct DNA-binding activity, we have shown that MBD3 can be recruited to methylated or non-methylated DNA by interacting with FBI-1. Because MBD3 is one of the subunits of the Mi-2/NuRD-HDAC complex, we tested whether the Mi-2/NuRD-HDAC complex is recruited to the *CDKN1A* promoter by interacting with FBI-1. Co-IP with the diagnostic subunits of the NuRD complex and western blotting showed that FBI-1 forms a complex composed of MBD3, Mi-2, MTA2, most notably in the cells co-transfected with FBI-1 and MBD3 (Figure 7A). DNMTs and HP1 proteins known to be associated with Mi-2/NuRD-HDAC were also detected in the immunoprecipitated complex (Figure 7A, right) (34).

We found that the molecular interaction between the co-repressor (NCoR, SMRT, BCoR) and FBI-1 was affected by MBD3, and the interaction between BCoR and FBI-1 was significantly increased. We suspected that such changes introduced by MBD3 might be important in gene silencing by DNA methylation. Co-IP with the BCoR antibody and western blotting showed that BCoR forms a complex with FBI-1, Mi-2/NuRD-HDAC complex, DNMTs and HP1 proteins (Figure 7B). By ectopic expression of BCoR, BCoR interaction with HDAC1, DNMT1 and DNMT3b is greatly enhanced, which may suggest more strong or direct interaction between BCoR and HDAC1or DNMTs (Figure 7B).

**Figure 8.**
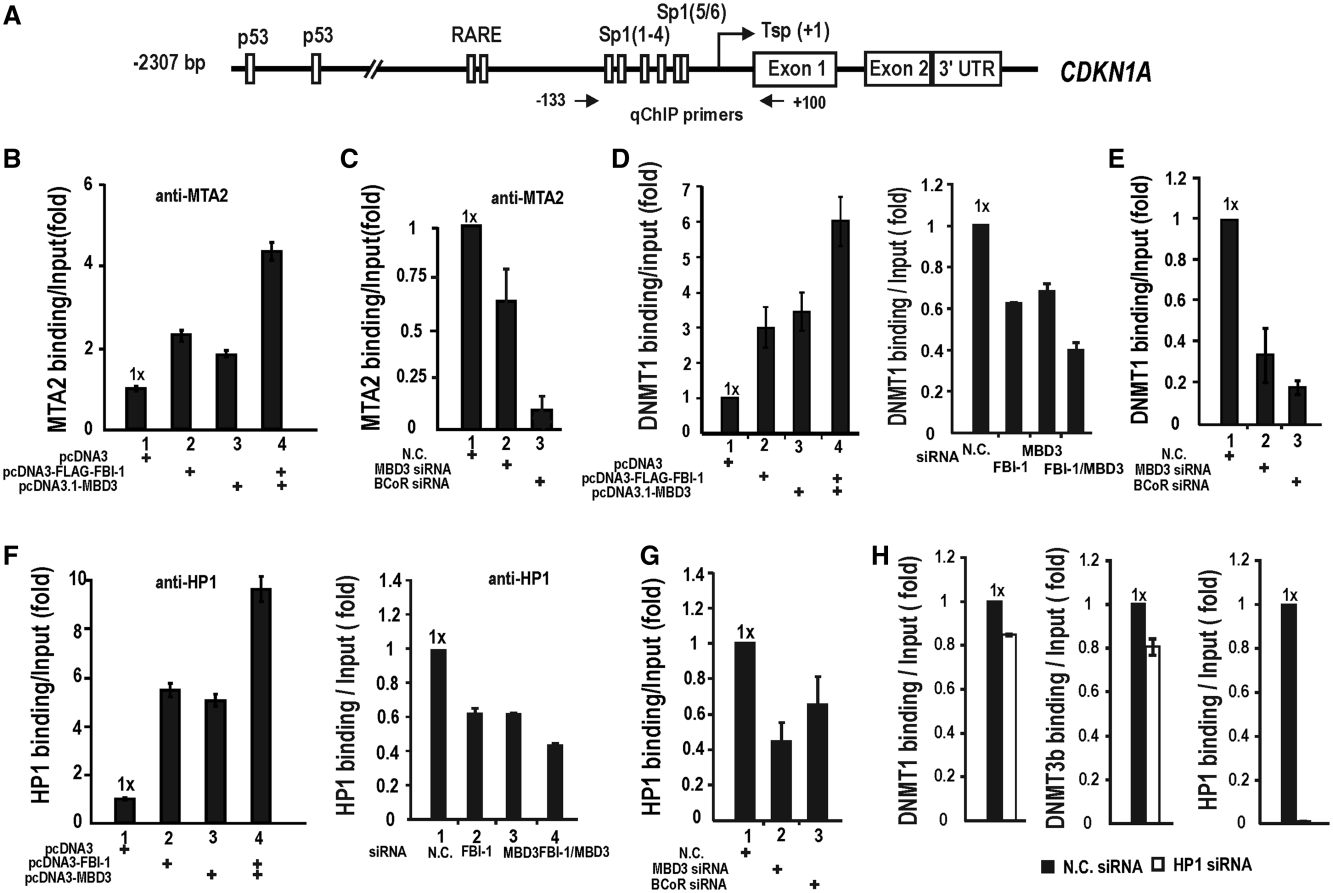
MBD3 and BCoR play critical role in the recruitment of Mi-2/NuRD-HDAC complex and subsequent binding of DNMTs and HP1. (**A**) Structure of human *CDKN1A* gene promoter. Arrows indicate the locations of qChIP PCR primer binding sites. (**B**) qChIP assays of Mi-2/NURD-HDAC complex (as tested for diagnostic subunit MTA2) binding at the endogenous *CDKN1A* promoter of the cells transfected with FBI-1 or/and MBD3. (**C**) qChIP assays of the Mi-2/NuRD-HDAC complex binding at the endogenous *CDKN1A* promoter after knockdown of MBD3 or BCoR. Knockdown of MBD3 or BCoR expression decrease Mi-2/NuRD-HDAC complex binding. (**D**) qChIP assays of DNMT1 binding in HEK293 cells. HEK293 cells were transfected with ectopic expression vectors or knockdown siRNA of MBD3 or/and FBI-1. Chromatin was fixed and DNMT binding was analyzed by qChIP. (**E**) qChIP assays of the DNMT1 binding at the endogenous *CDKN1A* promoter after knockdown of MBD3 or BCoR. Knockdown of MBD3 or BCoR expression potently decrease DNMT recruitment. (**F**) qChIP assays of HP1 binding to the endogenous *CDKN1A* promoter in HEK29A cells, in the presence of ectopic MBD3, FBI-1 or siRNA against MBD3 or BCoR. (**G**) qChIP assays of the HP1 binding at the endogenous *CDKN1A* promoter after knockdown of MBD3 or BCoR. Knockdown of MBD3 or BCoR expression decrease HP1 recruitment. (**H**) qChIP assays of DNMT1 or 3b binding. HEK293 cells were transfected with HP1 siRNA and analyzed for DNMTs binding.

We tested whether the Mi-2/NuRD-HDAC complex was recruited by FBI-1 to the *CDKN1A* promoter using ChIP assays with the anti-MTA2 and anti-DNMTs antibodies. MTA2 binding was increased by MBD3, and FBI-1, but most significantly by MBD3/FBI-1 co-expression (Figure 8A and B). Knock-down of MBD3 expression resulted in decreased MTA2 binding, suggesting that the NuRD complex is recruited to the *CDKN1A* promoter through the FBI-1–MBD3 interaction. Interestingly, knock-down of BCoR also resulted in a drastical decrease in MTA2 binding (Figure 8C). Because the interaction between FBI-1 and MBD3 significantly increases BCoR binding, BCoR may be important in the recruitment of Mi-2/NuRD-HDAC or BCoR may be a critical subunit of the Mi-2/NuRD-HDAC complex assembly as suggested by co-IP of BCoR and Mi-2/NuRD-HDAC complex (Figure 7B).

Furthermore, by ChIP assays, we investigated whether the recruitment of the DNMTs and HP1 associated with Mi-2/NuRD-HDAC complex is also increased by ectopic FBI-1 or/and MBD3. For DNMT1 and 3b, ectopic FBI-1 or MBD3 increased DNMT binding by 3- to 5-fold and co-expression of the two proteins increased DNMT binding by 6- to 8-fold. Knock-down of MBD3 or FBI-1 resulted in a 30–40% decrease in DNMT binding and knock-down of the two proteins resulted in a 60% decrease in DNMT binding (Figure 8D; Supplementary Figure S4A and B). In particular, knock-down of BCoR mRNA potently decreased DNMTs binding. We observed a similar binding pattern for HP1 (Figure 8F). In the presence of MBD3 and/or FBI-1, HP1 binding is increased in a pattern similar to the Mi-2/NuRD-HDAC complex and DNMT binding (Figure 8B–D). On knock-down of MBD3 or BCoR mRNA, HP1 binding is decreased (Figure 8G) and suggests that recruitment of the Mi-2/NuRD-HDAC complex is important in DNMT and HP1 binding.

HP1 and DNMTs were shown to form a complex (35,36). It was proposed that the complex is recruited to the regulatory DNA region by the HP1-H3K9-Me3 interaction and methylated DNA (37). However, knock-down of HP1 mRNA showed only weak effects on DNMT binding (Figure 8H) and suggested the importance of the DNMTs associated with the Mi-2/NuRD-HDAC complex in methylation of the promoter DNA. While HP1 binding is affected by FBI-1 and/or MBD3, knock-down of HP1 mRNA does not affect FBI-1 binding and recruitment of MBD3, and Mi-2/NuRD-HDAC complex (Supplementary Figure S5A and B). Interestingly, knock-down of HP1 mRNA resulted in increase in acetylated histones H3 and H4, H3K4-Me3 and decrease in H3K9-Me3, suggesting transcription repressor role of HP1, but how HP1 affects histone modifications is unknown.

The changes in binding patterns for the Mi-2/NuRD-HDAC complex, DNMT1/3b and HP1 by ectopic expression or knock-down of FBI-1 and/or MBD3 mRNA are well reflected by the changes in epigenetic histone marks for the transcriptionally active or silent states. While ectopic FBI-1 and/or MDB3 increases the amount of the transcription repression histone mark H3K9-Me3, knock-down of FBI-1 and/or MBD3 increased Ac-H3, Ac-H4 and H3K4-Me3, indicating transcription derepression (Supplementary Figure S4C and D). These results suggest that FBI-1, BCoR and MBD3 are important in binding of the Mi-2/NuRD-HDAC complex, DNMTs and HP1, which are important in epigenetic histone modification, promoter DNA methylation and heterochromatin formation around the *CDKN1A* promoter.

### MBD3 and FBI-1 repress transcription of *CDKN1A* by histone modification and DNA methylation of the proximal promoter in primary HDFn cells

**Figure 9.**
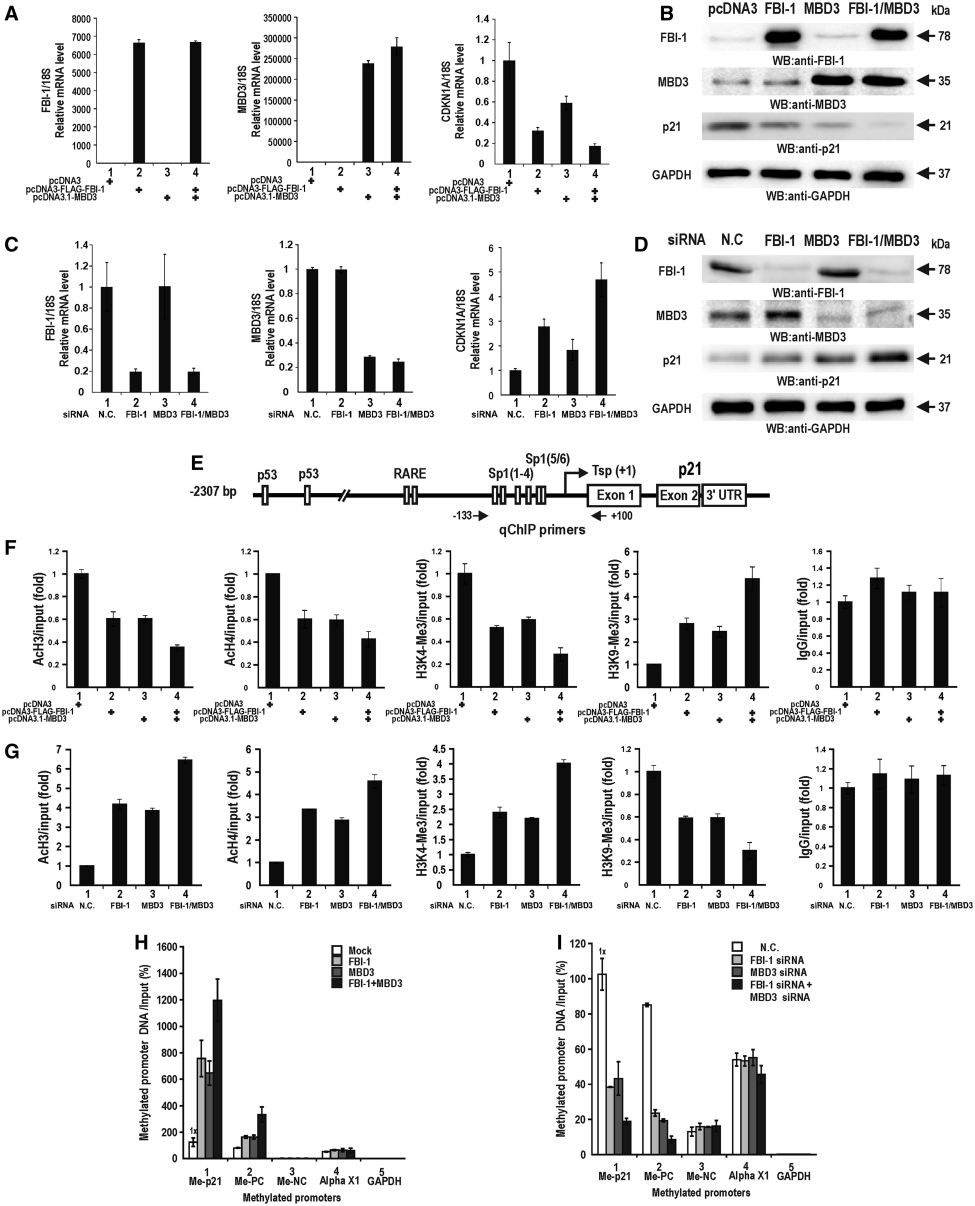
MBD3 and FBI-1 repress the transcription of *CDKN1A* by histone modification and DNA methylation in primary human HDFn cells. (**A** and **B**) RT-qPCR and western blot analysis of p21 expression in HDFn cells transfected with FBI-1 and/or MBD3 expression vectors. Ectopic FBI-1 and/or MBD3 decrease endogenous p21 expression. (**C** and **D**) RT-qPCR and western blot analysis of p21 expression in HDFn cells transfected with siRNA designed to knock down endogenous FBI-1 and/or MBD3 expression. Knock-down of FBI-1 and/or MBD3 increases endogenous p21 mRNA expression. (**E**) Structure of the human CDKN1A gene promoter. Arrows indicate the locations of the ChIP PCR primer-binding sites. (**F**) qChIP analysis of histone modifications at the proximal *CDKN1A* promoter caused by FBI-1 and/or MBD3 expression. Histone marks indicating active transcription (acetylated H3 and H4, H3K4-Me3) and repressed transcription (H3K9-Me3) were tested. The negative control was IgG. (**G**) qChIP analysis of histone modifications at the proximal promoter of the endogenous *CDKN1A* gene by knock-down of endogenous FBI-1 and/or MBD3 expression. The negative control was IgG. (**H** and **I**) Me-DIP assay of DNA methylation at the proximal *CDKN1A* promoter after ectopic expression or knock-down of FBI-1 and/or MBD3. Me-PC, methylation positive control; Me-NC, methylation negative control; GAPDH, GAPDH promoter; Alpha X1, X-linked alpha-satellites.

We examined whether the epigenetic silencing of CDKN1A by MBD3 and FBI-1 that we observed in various cancer and immortalized cells could also be possible in primary human cells such as HDFn cells. MBD3 and/or FBI-1 affected the transcriptional repression of endogenous *CDKN1A* in HDFn cells. FBI-1 or MBD3 alone repressed the expression of p21. However, when FBI-1 and MBD3 were co-transfected, strong repression of p21 was observed at both mRNA and protein levels (Figure 9A and B). Knock-down of FBI-1 and/or MBD3 resulted in the derepression of p21 expression (Figure 9C and D).

We also examined the changes in epigenetic histone marks around the proximal promoter of *CDKN1A* caused by ectopic FBI-1 and MBD3 expression or knock-down of FBI-1 and MBD3 mRNA. The two proteins decreased indicators of active transcription such as acetylated histones H3 and H4 and the trimethylated histone H3K4 (indicators of active transcription) while increasing the trimethylated histone H3K9, indicating transcriptional repression (Figure 9F and G).

Me-DIP assays of the endogenous *CDKN1A* promoter in HDFn cells showed that MBD3 and FBI-1 each increased methylation levels by >7-fold. In combination, they increased methylation by >12-fold; the two factors had no effect on the methylation status of positive or negative control promoters (Figure 9H). Knock-down of MBD3 and/or FBI-1 mRNA (Figure 9C) caused a significant decrease in DNA methylation of the *CDKN1A* proximal promoter and had no significant effect on the control promoters (Figure 9I). This result suggests that transcription of the *CDKN1A* proximal promoter might be repressed by DNA methylation of the complex containing FBI-1 and MBD3 in HDFn cells.

## DISCUSSION

FBI-1 is a proto-oncogenic transcription factor that is overexpressed in various human cancer tissues. It represses the transcription of *ARF* and *CDKN1A*, which play critical roles in cellular transformation, cell proliferation and oncogenesis (1–3). Tumour-suppressor genes like *CDKN1A* are often repressed in cancer tissues and remain in a repressed state during successive stages of cell proliferation. Previously, we demonstrated that FBI-1 interacts with co-repressor/HDAC complexes to repress the transcription of *CDKN1A* by histone deacetylation (1). Studies of the methylation status of the *CDKN1A* promoter in bone marrow cells in 124 patients with acute lymphoblastic leukaemia showed hypermethylation in 41% (51 of 124) of these patients. Hypermethylation within the promoter strongly correlated with decreased *CDKN1A* mRNA expression in tumour cells and such patients showed poor prognoses (31).

**Figure 10.**
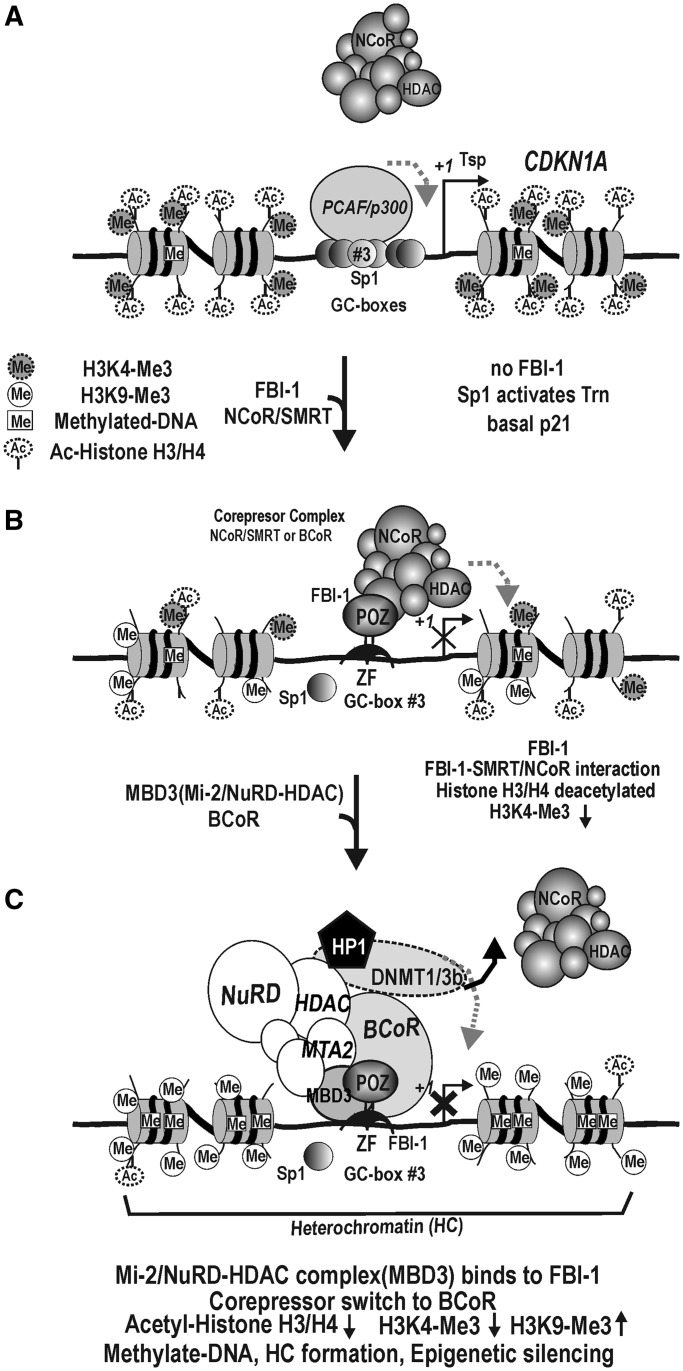
Hypothetical model of epigenetic silencing of the *CDKN1A* gene by FBI-1, MBD3 and BCoR, and DNA methylation by DNMT1/3b. (**A**) In normal cells with no or low FBI-1, transcription of *CDKN1A* is mainly regulated by Sp1 family proteins acting on GC-boxes of the proximal promoter, and cells express basal levels of p21. PCAF/p300, co-activator with HAT activity. Histones are acetylated and rich in H3K4-Me3. Few CpG are methylated. (**B**) With a high level of FBI-1, FBI-1 binds to the FRE (GC-box #3) critical in transcription initiation and activation, and interacts with a co-repressor (SMRT/NCoR) to repress transcription by deacetylation of histones H3 and H4, demethylation of H3K4-Me3 and methylation of H3K9. In this case, SMRT/NCoR competes with BCoR to interact with FBI-1. (**C**) In the presence of MBD3 and FBI-1, MBD3 binds to the zinc-finger domain of FBI-1 and recruits the Mi-2/NuRD-HDAC complex and directs FBI-1 to interact primarily with BCoR co-repressor. MBD3 and BCoR play critical roles in the recruitment of the Mi-2/NuRD-HDAC complex. Eventually, DNMT1/3b and HP1 proteins associated with the NuRD complex are recruited to methylate the promoter DNA, which forms a heterochromatin structure in the region.

We have previously shown that, to repress the transcription of *CDKN1A*, FBI-1 binds to the proximal promoter Sp1 binding GC-box #3 (or FRE), which is critical in transcription initiation and activation by interaction with Sp1 and by p53 bound at the distal regulatory elements of *CDKN1A* (1,38). The transcription of *CDKN1A* is derepressed by both TSA and 5-aza-2′-deoxycitidine, suggesting that the gene is repressed by a mechanism involving histone deacetylation and DNA methylation. We suspected that FBI-1 might also play a role in establishing and maintaining epigenetically silent *CDKN1A* by DNA methylation in cancer cells. We also found that FBI-1 interacts with MBD3 and that the molecular events initiated by the interaction are important in the epigenetic silencing of *CDKN1A* by DNA methylation (Figure 10, hypothetical model).

MBD family proteins are known to bind to methylated DNA and repress transcription, with the exception of MBD3, which is one of components of the Mi-2/NuRD-HDAC complex. MBD3 was shown to act as a transcriptional repressor by recruiting HDACs and DNMTs, which are important in oncogenic transformation and cell proliferation (20,39). An accepted role of MBD3 in transcriptional repression is by histone deacetylation by HDAC1 and HDAC2 associated with the Mi-2/NuRD-HDAC complex [(18) and references therein]. Recently, MBD3 was shown to be associated with the *CDKN1A* promoter and was dissociated from the promoter on TSA treatment; the functional significance of MBD3 binding remained unknown [(19) and references therein; (21)].

Previously, we found that FBI-1 interacts with the co-repressors SMRT, NCoR and SIN3A to repress transcription via histone modifications around the proximal promoter region. MBD3 enhanced transcription of the *CDKN1A* gene by FBI-1 (1). Initially, we suspected that additional recruitment of the co-repressors might be responsible for the increased repression. However, we found that binding of the above-mentioned co-repressors to the proximal promoter region was decreased. Intrigued by this finding, we investigated and successfully showed that, by interacting with FBI-1, MBD3 is targeted to the FRE of the proximal *CDKN1A* promoter and enhances transcription repression by FBI-1. MBD3 recruited by FBI-1 controls which co-repressor interacts with FBI-1. Although FBI-1 can interact with NCoR, SMRT and BCoR, MBD3 leads FBI-1 to interact primarily with BCoR to repress the transcription of *CDKN1A* by DNA methylation. The transition of interacting co-repressor with BCoR increased the binding of the Mi-2/NuRD-HDAC complex (Figure 7B), resulting in the deacetylation of histones, demethylation of histone H3K4 and an increase in trimethylation of histone H3K9. BCoR plays a critical role in Mi-2/NuRD-HDAC complex recruitment (Figure 7B and 8C). In addition, DNMTs and HP1 proteins associated with the Mi-1/NuRD-HDAC complex are recruited to methylate DNA and to maintain epigenetically repressed states (Figure 10). Accordingly, by interacting with FBI-1, MBD3 may be engaged in both *de novo* methylation and also maintenance of the methylated *CDKN1A* promoter by recruitment of DNMTs. It was reported that DNMT1 represses the *CDKN1A* promoter. But the mechanism of DNMT1 action on the promoter remained unknown (25,26). Our study showed how DNMTs are recruited to the *CDKN1A* promoter region and can repress transcription by DNA methylation in the presence of FBI-1, MBD3 and BCoR.

Interestingly, FBI-1 could bind both non-methylated and methylated DNA, a property similar to that of another methylated DNA–binding protein, KAISO. FBI-1 not only represses transcription of its target genes by histone deacetylation, but also it can silence its target genes by DNA methylation involving MBD3 and BCoR. By interacting with MBD3 and recruiting BCoR to the FBI-1-MBD3 complex to the *CDKN1A* promoter, FBI-1 can repress *CDKN1A* expression and stimulate cell proliferation. We have previously demonstrated that FBI-1 regulates the p53 pathway genes, which are important in regulating cell proliferation. ChIP-on-ChIP analysis of the FBI-1 target genes in HepG2 cells revealed that numerous genes important in cell metabolism and cell proliferation are targets of FBI-1 (40). Accordingly, these genes, either methylated or non-methylated, are likely regulated by FBI-1, MBD3 and BCoR in a manner similar to *CDKN1A*.

The epigenetic gene silencing observed for FBI-1 might also be applicable to other POK family transcription factors. For example, proto-oncoprotein BCL6 was shown to interact with the co-repressors SMRT, NCoR and BCoR (BCL6 co-repressor) in a mutually exclusive fashion (41). The BCoR complex contains polycomb group protein (PcG), E3 ubiquitin ligase and histone H3K36 demethylase, suggesting that BCoR may use epigenetic modification to silence gene expression (42). BCL6 also interacts with the MBD3-Mi-2/NuRD-HDAC complex to repress transcription of plasma-specific genes to determine the cell fate of B lymphocytes (43). BCoR was shown to regulate mesenchymal stem cell function by an epigenetic mechanism (44). The molecular mechanism we found here may give insight into the role of BCL6 and its interaction with BCoR in the epigenetic regulation of BCL6 target genes, including *CDKN1A* and *TP53*. Interestingly, it was demonstrated that BTB/POZ protein HIC1 target genes are differentially regulated by CtBP and NuRD via an acetylation/SUMOylation switch. HIC1 participates in the complex regulation of the genes crucial for cell growth and survival through gene-specific co-repressor recruitment (45).

Although involvement of BCoR was not illustrated, a similar epigenetic silencing mechanism involving Mi-2/NuRD-HDAC complex seems to be used by the oncoprotein, PML-RARα. PML-RARα binds and recruits Mi-2/NuRD-HDAC to the tumour-suppressor gene RARβ2 to silence the RARβ2 promoter. The Mi-2/NuRD-HDAC complex plays a direct role in the aberrant gene repression and transmission of epigenetic repressive marks in acute promyelocytic leukaemia (APL) (39). The Mi-2/NuRD-HDAC complex facilitates polycomb binding and histone methylation at lysine 27. Knock-down of the Mi-2/NuRD-HDAC complex in leukaemic cells not only prevented histone deacetylation and chromatin compaction but also impaired DNA and histone methylation and stable silencing. Mi-2/NuRD-HDAC plays an important role in the establishment of altered epigenetic marks in APL.

Overall, our studies suggest that FBI-1 recruits BCoR and the Mi-2/NuRD-HDAC complex to the *CDKN1A* promoter through interaction with MBD3, which is followed by histone modifications, DNA methylation, HP1 binding and epigenetic silencing of *CDKN1A*. The molecular events that lead to promoter DNA methylation of *CDKN1A* may be important in the oncogenic ability of FBI-1 and cell proliferation.

## SUPPLEMENTARY DATA

Supplementary Data are available at NAR Online: Supplementary Table 1, Supplementary Figures 1–9 and Supplementary Materials and Methods.

Supplementary Data
