# Supplementary material for: The proto-oncoprotein FBI-1 interacts with MBD3 to recruit the Mi-2/NuRD-HDAC complex and BCoR and to silence p21WAF/CDKN1A by DNA methylation
Source: Nucleic Acids Res. 2013 May 8;41(13):6403–20. doi: 10.1093/nar/gkt359 (PMC3711425; doi:10.1093/nar/gkt359)
Supplement: Supplementary Data [file supp_41_13_6403__index.html]

The proto-oncoprotein FBI-1 interacts with MBD3 to recruit the Mi-2/NuRD-HDAC complex and BCoR and to silence p21WAF/CDKN1A by DNA methylation — Supplementary Data 

# The proto-oncoprotein FBI-1 interacts with MBD3 to recruit the Mi-2/NuRD-HDAC complex and BCoR and to silence *p21WAF/CDKN1A* by DNA methylation

## Supplementary Data

files

**Files in this Data Supplement:**

- Supplementary Data - pdf file
