# Supplementary material for: The proto-oncoprotein FBI-1 interacts with MBD3 to recruit the Mi-2/NuRD-HDAC complex and BCoR and to silence p21WAF/CDKN1A by DNA methylation
Source: Nucleic Acids Res. 2013 May 8;41(13):6403–20. doi: 10.1093/nar/gkt359 (PMC3711425; doi:10.1093/nar/gkt359)
Supplement: Supplementary Data [file supp_gkt359_Supplemental_Data_Upload_20130426_OUP_edited.pdf]

SUPPLEMENTARY INFORMATION

The proto-oncoprotein FBI-1 interacts with MBD3 to recruit the Mi-2/NuRD-HDAC complex and BCoR and to silence *p21WAF/CDKN1A* by DNA methylation

Won-Il Choi, Bu-Nam Jeon, Jae-Hyeon Yoon, Dong-In Koh, Myung-Hwa Kim, Mi-Young Yu, Kyung-Mi Lee, Youngsoo Kim, Kyunggon Kim, Sujin Susanne Hur, Choong-Eun Lee, Kyung-Sup Kim, and Man-Wook Hur*

SUPPLEMENTAL MATERIALS & MATHODS

SUPPLEMENATRY FIGURE & LEGENDS

SUPPLEMENTARY MATERIALS AND METHODS.

Cell culture/stable cell line

HEK293 cells were cultured in Dulbecco's modified eagle medium (DMEM, Gibco-BRL, Gaithersburg, MD) supplemented with 10% FBS at 37°C, 5% CO_2. Primary human dermal fibroblast neonatal (HDFn) cells were cultured in Medium 106 (Gibco-BRL, Gaithersburg, MD) supplemented with Low Serum Growth Supplement (LSGS; Gibco-BRL, Gaithersburg, MD) in the absence of antibiotics and antimycotics at 37 °C, 5% CO_2. Drosophila SL2 cells were cultured in Schneider’s Drosophila medium (Gibco-BRL, Gaithersburg, MD) supplemented with 10% FBS.

Stable HEK293TRex-FBI-1 cells inducible by doxycycline were used to express Flag-FBI-1. The stable cells were cultured in medium containing 1 μg/ml doxycycline for the intended period of time.

Inducible HEK293Trex-FLAG-FBI-1 cell preparation

Nuclear extract was prepared from the HEK293TrexFlag-FBI-1 cells that stably over express FBI-1. To prepare stable cell, FLAG tagged FBI-1 in the pcDNA5/FRT/TO expression vector and pOG44 were co-transfected into the Flp-In^{TM}T-RE$_X$^{TM}293T cells (Invitrogen, Carlsbad, CA). T-RE$_X$-FBI-1 cells were selected by blasticidin and hygromycin and FBI-1 expression was induced by doxycycline. To isolate nuclear protein complexes that interact with FBI-1 from doxycycline inducible stable HEK293-TREx-FLAG FBI-1 cells, FBI-1 expression was induced overnight and the nuclei were separated by glycerol gradient centrifugation.

Western blot analysis

HEK293 Flp-In^{TM} T-REx^{TM} FBI-1 overexpressing FBI-1 cells were harvested and lysed in TEN buffer (10 mM Tris-HCl pH 8.0, 1 mM EDTA, and 0.1 M NaCl). Cell extracts (40 μg) were separated by a 10% SDS-PAGE. Proteins were transferred onto an Immun-Blot^{TM} PVDF membrane (Bio-Rad, Richmond, CA) and blocked with 5% skim milk (BD Biosciences, Oxford, UK) in TBST (20 mM Tris-HCl pH 7.5, 140 mM NaCl, and 0.001% Tween 20) for 10 min. Blotted membranes were incubated with antibody against FBI-1 (Sigma, St. Louis, MO) at 4°C for overnight. Membranes were washed three times with TBST for 10 min each and incubated with horseradish peroxidase-conjugated anti-goat IgG (Santa Cruz Biotechnology, Santa Cruz, CA) antibody. Blots were washed with TBST three times and developed with ECL solution (PerkinElmer, Foster City, CA).

Preparation of nuclear extract

HEK293 Flp-In^{TM} T-REx^{TM} FBI-1 stable cells were harvested from cell culture media by

centrifugation for 10 min at 15.4× *g* (2,000 rpm, Eppendorf centrifuge 5810R, Germany). The cells were resuspended in five packed cell pellet volumes of buffer A (10 mM HEPES (pH 7.9 at 4°C), 1.5 mM $MgCl_2$, 10 mM KC1 and 0.5 mM DTT) and lysed by 10 strokes of a glass Dounce homogeniser (loose type, Wheaton, Millville, NJ). The homogenate was centrifuged for 10 min at 15.4× *g* to collect the nuclear pellet. The pellet was resuspended in buffer C [20 mM HEPES (pH 7.9), 25% (v/v) glycerol, 0.42 M NaCl, 1.5 mM $MgCl_2$, 0.2 mM EDTA, 0.5 mM phenylmethylsulfonyl fluoride (PMSF), and 0.5 mM DTT] with a glass Dounce homogeniser (Tight type, Wheaton, Millville, NJ). The resulting suspension was stirred gently with a magnetic stirring bar for 30 min and then centrifuged for 30 min at 25,000 × *g*. The supernatant was dialysed against 50 volumes of buffer D [20 mM HEPES (pH 7.9), 20% (v/v) glycerol, 0.15 M NaCl, 0.2 mM EDTA, 0.5 mM phenylmethylsulfonyl fluoride (PMSF), and 0.5 mM DTT] for 5 h. The dialysate was centrifuged at 25,000 × *g* for 20 min and the resulting precipitate was discarded. The supernatant, containing nuclear extract, was frozen as aliquots in liquid nitrogen and stored at -80°C.

GST fusion protein purification, *in vitro* transcription and translation

GST, GST-POZFBI-1, and GST-ZFFBI-1 proteins were prepared from *E. coli* BL21 (DE3) transformed with the pGEX4T series GST, GST-POZFBI-1, and GST-ZFFBI-1 protein expression plasmids. *E. coli* cultures were induced with 0.5 mM isopropyl-1-thio-D-galactopyranoside (IPTG) for 4 h at 37°C. The cells were lysed in a buffer containing 1× PBS, 1 mM PMSF, 2 mM EDTA, and 0.2 mg/ml lysozyme, and sonicated 3-5 times at 0.5 cycle and 50% amplitude (Dr. Hielscher GmbH, Germany) to prepare lysates. The recombinant proteins were purified with glutathione-agarose 4 beads by affinity chromatography (Peptron, Daejeon, Korea). The purified proteins were resolved with 12% SDS-PAGE to quantitate and assess purity. Aliquots of the protein-agarose bead complex were used in GST-fusion protein pull down assays.

MBD3 was prepared *in vitro* by incubating 1 μg of pcDNA3.0 expression plasmid DNA with TNT Quick-coupled Transcription/Translation Extract (Promega, Madison, WI) containing 40 μl of TNT Quick Master Mix and 2 μl of [^{35}S]-methionine (1175.0 Ci/mol, PerkinElmer) at 30°C for 90 min. Polypeptide expression level was analysed by running 3 μl of the total mixture on a 10% SDS-PAGE.

GST fusion protein pull-down assays

The purified GST fusion proteins (5 μg) were incubated with GSH-agarose (Sigma, St. Louis, MO) for 1 h in HEMG buffer [40 mM HEPES, pH 7.9, 100 mM KCl, 0.2 mM EDTA, 5 mM $MgCl_2$, 0.1% Nonidet P-40, 10% glycerol, 1.5 mM dithiothreitol, and 1 tablet/50 ml of a protease inhibitor mixture (Roche, Mannheim, Germany)] at 4°C for 1 h. After the agarose-GST protein complexes were washed

three times with 1 ml of cold HEMG buffer, 10 μl of the *in vitro* translated MBD3 was added and incubated in HEMG buffer at 4°C for 4 h. The reaction mixtures were centrifuged at 3,000 × *g* at 4°C, the supernatants were removed, and the pellets were washed five times with cold HEMG buffer. The bound proteins were separated by a 10% SDS-PAGE. The SDS-PAGE gel was dried and exposed to X-ray film using an image-intensifying screen (Kodak, Rochester, NY).

Immunofluorescence assay

HeLa cells grown on coverslips (Sunshine Works, Seoul, Korea) were washed with cold PBS and fixed in 97:3 cold methanol/formaldehyde for 20 min at -20°C. Cells were permeabilised in 0.2% Triton X-100, washed with PBS, and were incubated in 5% normal horse serum for blocking. Cells were incubated with the mouse anti-FLAG primary antibody for 2 h at room temperature, rinsed, and further incubated with FITC-conjugated anti-mouse IgG secondary antibody for 1 h at room temperature. For double staining, cells were fixed again with 3.7% formaldehyde, incubated with blocking solution, and incubated with a different primary antibody (rabbit anti-His antibody), rinsed, and incubated with secondary antibody (anti-rabbit antibody conjugated with Rhodamine. Cells were then washed, and a final wash was performed in a solution containing 1 mg/ml 4, 6-diamidino-2-phenylindole. Cells were mounted on glass slides with mounting medium [90% (v/v) glycerol, 1 mg/ml p-phenylenediamine, and 0.02% sodium azide] and examined with LSM 510 confocal laser scanning microscope (Carl Zeiss, Oberkochen, Germany).

Oligonucleotide pull-down assay

HEK293 cells transfected with pcDNA3.0 or pcDNA3-FLAG-FBI-1 expression vector were cultured for 48 h. HEK293 cells were lysed in HKMG buffer (10 mM HEPES, pH 7.9, 100 mM KCl, 5 mMMgCl2, 10% glycerol, 1 mM dithiothreitol, and 0.5% Nonidet P-40). Cellular extracts were pre-cleared with streptavidin-agarose beads (Thermo, Rockford, IL) for 1 h, and incubated with 1 μg of biotin labeled FRE and Me-FRE (see Materials and Methods) for 16 h. To collect proteins bound to FRE or Me-FRE, the mixtures were incubated with streptavidin agarose beads for 2 h, washed with HKMG buffer, and precipitated by centrifugation. The precipitates were separated by SDS-PAGE and was analyzed by Western blotting assay using anti-FBI-1, anti-MBD3 and anti-GAPDH antibodies.

EMSA (electro-mobility shift assay)

EMSAs were carried out as described previously (1). Oligonucleotide sequences used are as follows; FRE (forward, 5’-GATCCGAGCGCGGGTCCCGCCTC-3’; reverse, 5’-GATCGAGGCGGGACCCGCGCTCG-3’). Methylated oligonucleotide probes were synthesized by

Integrated DNA Technologies (Coralville, IA). Methylated oligonucleotide probes used are as follows; Me-FRE (forward, 5'- GATC/5-Methyl-dC/GAG/5-Methyl-dC/G/5-Methyl-dC/GGGTCC/5-Methyl-dC/GCCTC-3'; reverse, 5'- GATCGAGG/5-Methyl-dC/GGGACC/5-Methyl-dC/G/5-Methyl-dC/GCT/5-Methyl-dC/G-3'). The FRE, and methylated FRE probes were labeled using ^{32}P-γ–ATP and polynucleotide kinase. Each EMSA binding reaction was carried out for 30 min in 20 μl of binding buffer (10 mM HEPES pH 7.9, 60 mM KCl, 0.005 mM $ZnCl_2$, 1 mM dithiothreitol, 1% BSA, and 7% glycerol) and 200 ng recombinant GST-ZF FBI-1. The protein-DNA complexes were resolved with 4% non-denaturing PAGE at room temperature in 0.5X TBE (89mM TBE, pH 8.3) at 150 V for 2 h. The gels were exposed to X-ray film with a Kodak intensifying screen.

Mammalian two-hybrid reporter assays

Monkey kidney cells (CV-1) were grown in DMEM supplemented with 10% FBS (Invitrogen, Carlsbad, CA). The cells were inoculated into 6-well culture dishes at a density of 5×10^5 cells/well. After the cells were grown for 16 h, they were transiently transfected with the pCMX-GAL4-ZFFBI-1 (bait), VP16 fusion protein expression plasmids (pCMX-VP16-BCoR, pCMX-VP16-NCoR, pCMX-VP16-mSin3A, and pCMX-VP16-SMRT), and the reporter plasmid (pG5-*Luc*) using the Lipofectamine reagent (Invitrogen, Carlsbad, CA). Cells were cultured for an additional 36 h. The cells were harvested and lysed in 100 μl of reporter lysis buffer (Promega, Madison, WI) and analysed for luciferase activity. Reporter assays were repeated 3-4 times and performed with 5 μl of cell extract, using 50 μl of luciferase assay reagent (Promega, Madison, WI) on a luminometer (Microplate Luminometer LB 96V, EG&G Berthold, Natick, MA). Luciferase activities were normalized with cotransfected β-galactosidase activity.

Knockdown of gene expression by siRNA

Two siRNA designed to knockdown each of the following genes expression, glyceraldehyde-3-phosphate dehydrogenase (GAPDH), FBI-1, MBD3, BCoR, and HP1 were purchased from Bioneer Inc. (Daejeon, Korea). siRNA (200 pmoles) was transfected into 6×10^5 HEK293 cells using Lipofectamin RNAiMAX (Invitrogen, Carlsbad, CA) and was transfected into 5×10^5 HDFn cells using the Neon Transfection System (Invitrogen, Carlsbad, CA). Total RNA was prepared from the cells using TRIzol (Invitrogen, Carlsbad, CA) and analysed for knockdown efficiency by RT-qPCR or semi-quantitative PCR. To test the knockdown efficiency at protein level, total cell extracts were prepared by lysis in RIPA buffer (50 mM Tris-HCl pH 8.0, 1% NP-40, 0.25% sodium deoxycholic acid, 150 mM NaCl, 1 mM EGTA and complete mini-protease cocktail). The cell lysates (50 μg) were analyzed by Western blotting analysis. Sequences of siRNA targeting GAPDH, FBI-1, MBD3, BCoR,

and HP1 mRNA are described Supplementary Table I.

Transcriptional analysis of the *CDKN1A*-Luc promoter

Two *CDKN1A*-Luc promoter reporter fusion plasmids (with -2.4 kb or -133 bp upstream regulatory sequence), pcDNA3-FBI-1 and pCMV-LacZ, were transiently transfected into HEK293 cells using Lipofectamine Plus reagent. After 48 h of incubation, cells were harvested and analysed for luciferase activity. The assays were repeated 3 times. The reporter activity was normalised with co-transfected β-galactosidase activity or protein concentration.

Two-dimensional gel electrophoresis

Isoelectric focusing (IEF) was performed using pre-cast immobilised pH gradient strips (24 cm, pH 3-10, linear, Amersham Biosciences, Uppsala, Sweden). 500 μg of proteins were solubilised in rehydration buffer (9 M urea, 2% CHAPS, 60 mM DTT, 0.5% pharmalyte, pH 3-10, 0.002% bromophenol blue) and protein samples were loaded on IPG strips and rehydrated overnight. The IEF gel was run for a total of 36 kVh during which the voltage was increased linearly from 100 to 8000 V over 6 h and then, maintained for 3 h at 8000 V. After IEF, strips were first equilibrated for 15 min in a reducing solution (50 mM Tris-HCl, pH 8.8, 6 M urea, 30% (v/v) glycerol, 2% (w/v) SDS, 1% (w/v) DTT), and then for a further 15 min in an alkylating solution, which was identical in make-up to the reducing solution except that 2.5% (w/v) iodoacetamide was substituted for DTT. A second gel electrophoresis was performed using a standard sodium dodecyl sulfate-polyacrylamide gel electrophoresis (SDS-PAGE) protocol, using the Ettan DALT 6 System (Amersham Biosciences, Uppsala, Sweden). SDS-PAGE was run on 12% polyacrylamide gel, and gels were visualised by silver staining.

In-gel digestion and mass spectrometry

Sliver-stained gel spots were excised, destained by reduction using a solution of 30 mM potassium ferricyanide/100 mM sodium thiosulfate, and washed with distilled water. The gel pieces were then incubated with 0.2 M NH_4HCO_3 for 20 min, dehydrated, shrunk with 100% acetonitrile twice, and dried by vacuum centrifugation. For in-gel digestion with trypsin, gel pieces were rehydrated in digestion buffer containing 0.05 M NH_4HCO_3 and 10 ng/μl of modified porcine trypsin (Promega, Madison, WI) at 4°C for 45 min. Excess supernatant was then removed, and the gel pieces were covered with 30 μℓ of 0.05 M NH_4HCO_3 buffer. Digestion was performed overnight at 37°C, and after in-gel tryptic digestion, tryptic peptides were extracted from the gel particles and trifluoroacetic acid (TFA) was added to a final concentraction of 0.1% to prevent additional digestion.

Samples were desalted using a GELoader tip (Eppendorf AG, Hamburg, Germany) and packed with POROS 20 R2 resin (Applied Biosystems Inc., Foster City, CA). Peptide binding and washing were performed using 0.1% TFA in water. To produce the MALDI sample matrix solution, α-cyano-4-hydroxy cinnamic acid was dissolved in a solution containing 70% acetonitrile and 0.1% TFA at concentration of 5 g/l. Elution was performed with 1 $\mu\ell$ of sample matrix solution and the eluted peptides were directly spotted onto the target plate.

Protein identification was carried out using peptide mass fingerprinting (PMF) and MS/MS analysis on the MALDI-TOF/TOF mass spectrometer system (ABI 4700 Proteomics Analyzer; Applied Biosystems, Foster City, CA). External calibrations using a 4700 calibration mixture (Applied Biosystems, Foster City, CA) in MS mode and glu-fibrinopeptide B fragment (m/z 1570.68) in MS/MS mode, respectively, were performed with a mass tolerance of 50 ppm. MS data and MS/MS data were acquired using 1000 and 3000 shots of a 355 nm Nd:YAG laser with a fixed intensity in positive ion reflector mode, respectively. In MS/MS analysis, CID was performed using air as the collision medium. The MS and MS/MS spectra were processed for protein identification with GPS Explorer™ software (Applied Biosystems, Foster City, CA) that adopted the MASCOT search algorithm (Matrix Science Ltd., Boston, MA) using the NCBI protein sequence database (http://www. ncbi.nlm.nih.gov/entrez) imbedded system.

SUPPLEMENTARY TABLE 1. NUCLEOTIDE SEQUENCES OF siRNA.

**Negative Control, SN-1013**	
**Sense**	**CCUACGCCACCAAUUUCGU(dTdT)**
**Anti Sense**	**ACGAAAUUGGUGGCGUAGG(dTdT)**
**HP1 #1 1025971**	
**Sense**	**UCUCAAACAGUGCCGAUGA(dTdT)**
**Anti Sense**	**UCAUCGGCACUGUUUGAGA(dTdT)**
**HP1 #2 1025966**	
**Sense**	**GCGCGUGGUUAAGGGACAA(dTdT)**
**Anti Sense**	**UUGUCCCUUAACCACGCGC(dTdT)**
**ZBTB7A #1 1165418**	
**Sense**	**CUCUGAGCGGACGUUAAAA(dTdT)**
**Anti Sense**	**UUUUAACGUCCGCUCAGAG(dTdT)**
**ZBTB7A #2 1165422**	
**Sense**	**CCUCGCAAUAAAACCAACU(dTdT)**
**Anti Sense**	**AGUUGGUUUUAUUGCGAGG(dTdT)**
**MBD3 #1 1092749**	
**Sense**	**GCUUCCCAUCUCCCUAGAA(dTdT)**
**Anti Sense**	**UUCUAGGGAGAUGGGAAGC(dTdT)**
**MBD3 #2 1092753**	
**Sense**	**CACAGUCGAGGCACGUCAU(dTdT)**
**Anti Sense**	**AUGACGUGCCUCGACUGUG(dTdT)**
**BCoR #1 1012083**	
**Sense**	**CGUUACCAAAGACGUCACA(dTdT)**
**Anti Sense**	**UGUGACGUCUUUGGUAACG(dTdT)**
**BCoR #2 1012084**	
**Sense**	**GACUAGACGCUUGAAGCAA(dTdT)**
**Anti Sense**	**UUGCUUCAAGCGUCUAGUC(dTdT)**

SUPPLEMENATRY FIGURES & LEGENDS

Supplemenatry Figure S1. Related to Figure 1. MBD3 is a component of the nuclear protein complex containing FLAG-FBI-1. (A) Control, 2D IEF/12% SDS-PAGE gel analysis of the immunoprecipitate. The nuclear extract prepared from HEK293-TREx control cells was immunoprecipitated using the anti-FLAG M2-Agarose antibody. (B) 2D gel of the immunoprecipitate of the nuclear protein complex prepared from the HEK293-TREx-FLAG-FBI-1 cells. Nuclear lysates were immunoprecipitated using the anti-FLAG M2-Agarose antibody and the immunoprecipitates were separated by 2D IEF (pH 3-10)/12% SDS-PAGE and stained with a silver staining solution. The spots that showed differential expression were isolated, digested with trypsin and subjected to MALDI-TOF mass spectrometry analysis. The black bordered circles were labelled with the name of the identified protein.

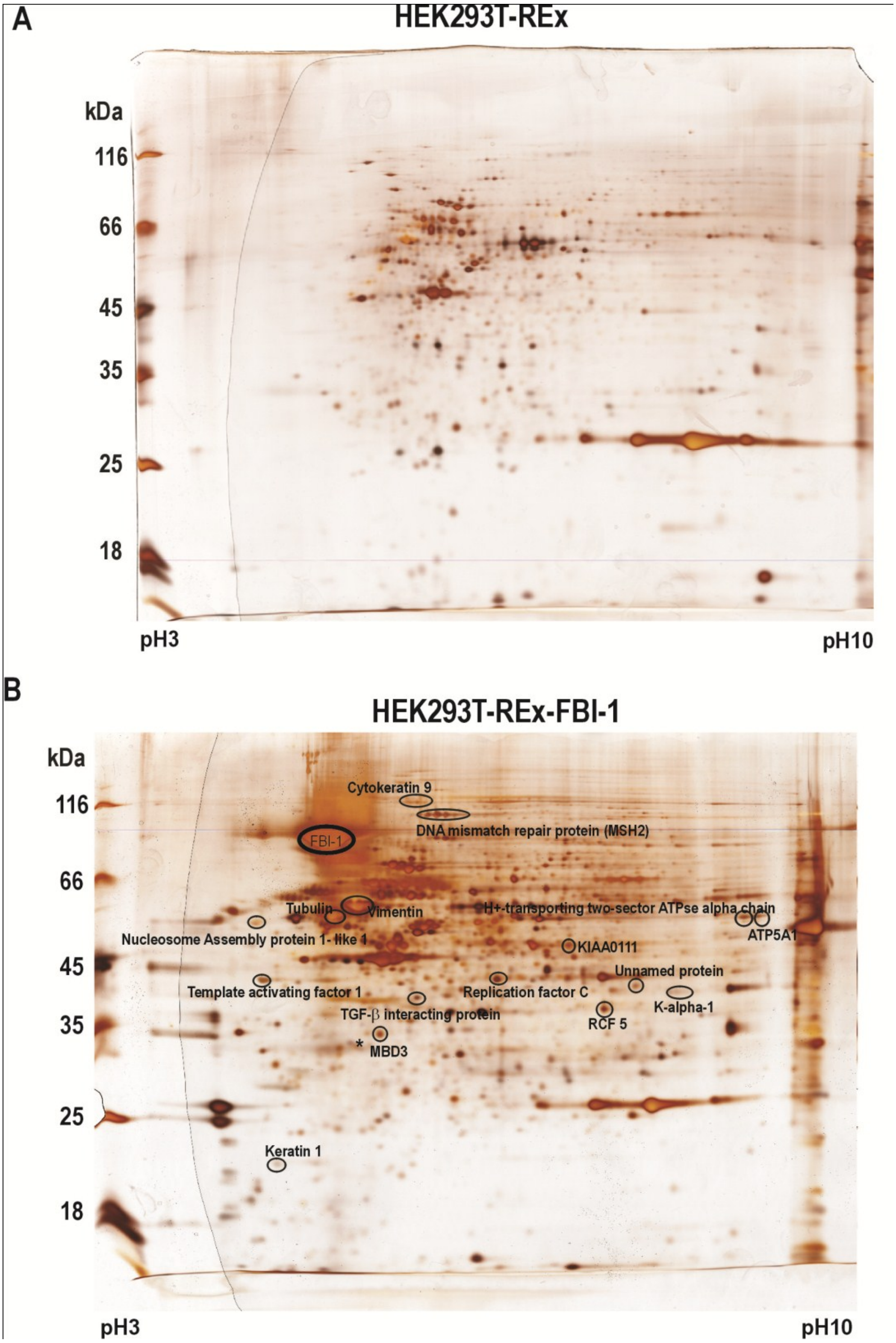

Supplementary Figure S1. ***Choi et al., 2012***

Supplemenatry Figure S2. Related to Figure 1. MALDI-TOF mass spectrometry analysis of the 34 kDa protein spot. (A) MALDI mass spectrum of the 34 kDa protein band that was immunoprecipitated with FLAG-FBI-1. (B) Peptide mass fingerprinting to identify the protein by MALDI-TOF mass spectrometry. Eight polypeptides that showed significant identity to MBD3 upon amino acid sequencing are listed. The 34 kDa protein was identified as MBD3 using a MASCOT database search.

A

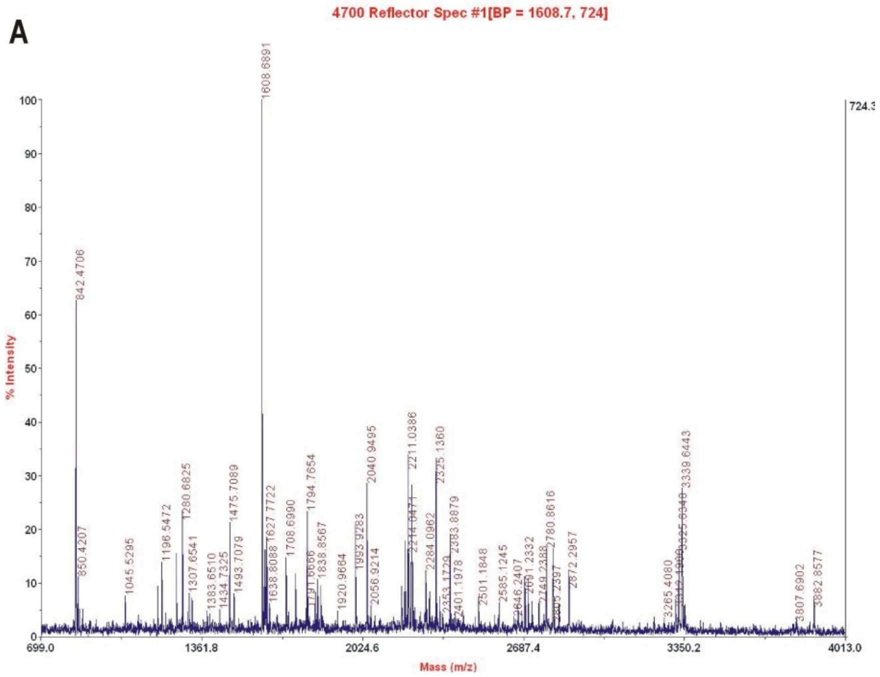

B

a.a. number sequenced	peptide sequence
20-33	YLGGSMDLSTFDFR
59-70	GKPDLNTALPVR
104-109	QLFWEK
110-125	KLSGLNAFDIAEELVK
171-184	NPGVWLNTTQPLCK
185-194	AFMVTDEDIR
196-205	QEELVQQVRK
206-224	RLEEALMADMLAHVEELAR

Supplementary Figure S2. ***Choi et al., 2012***

Supplementary Figure S3. Related to Figures 2E; 4E; 5H, I; 6F; 8. Semi-quantitative PCR analysis of knock-down of FBI-1, MBD3, BCoR, and HP1 mRNA expression by siRNA treatment. (A) Semi-quantitative RT-PCR analysis of p21 mRNA expression in the HEK293 cells transfected with various combinations of siRNA designed to knock-down endogenous FBI-1, MBD3 and HP1 mRNA expression. Knock-down of FBI-1, and/or MBD3 increases endogenous p21 mRNA expression. Knock-down of HP1 alone increases p21 mRNA expression. Knock-down of HP1 in combination with knock-down of both MBD3 and FBI-1 increases p21 mRNA expression. (B) Semi-quantitative RT-PCR analysis of mRNA expression after treatment with two different siRNA designed knock-down FBI-1, MBD3, BCoR, and HP1. mRNA are degraded effectively by siRNA. Each siRNA targeting FBI-1, MBD3, BCoR, or HP1 mRNA does not affect the mRNA levels of other mRNA. (C) Semi-quantitative RT-PCR analysis of mRNA expression after treatment with siRNA designed to knock-down FBI-1, MBD3, and BCoR.

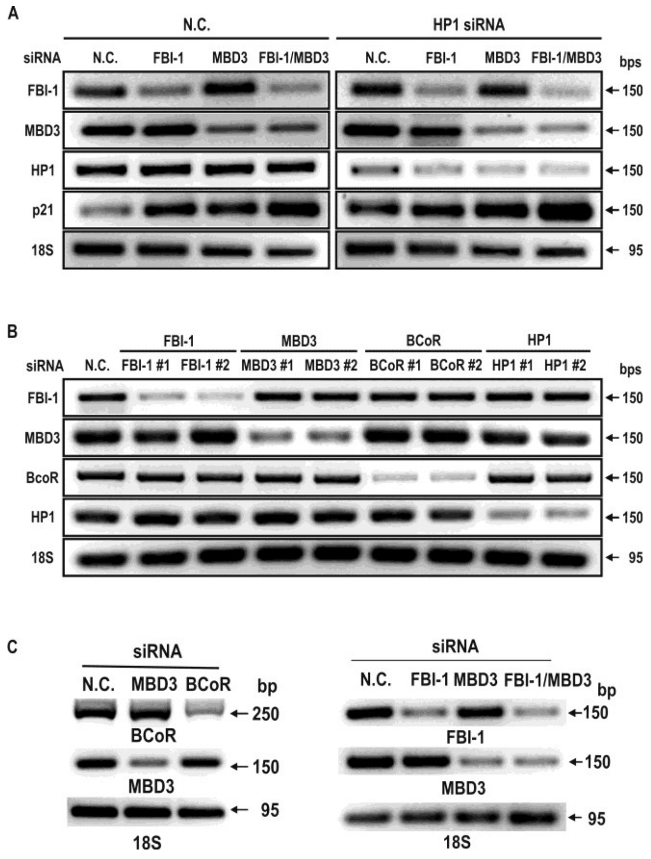

Supplementary Figure S3. ***Choi et al., 2012***

Supplementary Figure S4. Related to Figure 8. MBD3 and FBI-1 increase recruitment of DNMT3b at the proximal promoter of *p21WAF/CDKN1A* and significantly affect epigenetic histone marks. (A, B) qChIP assays of DNMT3b binding at the human *CDKN1A* gene promoter in HEK293 cells. HEK293 cells were transfected with ectopic expression vector or knockdown siRNA of MBD3 and/or FBI-1. Chromatin was fixed and DNMT binding was analyzed by qChIP. Knockdown of MBD3 or BCoR decreases DNMT3b binding. (C, D) qChIP assays of acetylated H3 and H4, H3K4-Me3, and H3K9-Me3 around the endogenous proximal *CDKN1A* promoter. HEK293 cells were transfected with FBI-1 and/or MBD3 expression vector or siRNA targeting FBI-1 and/or MBD3.

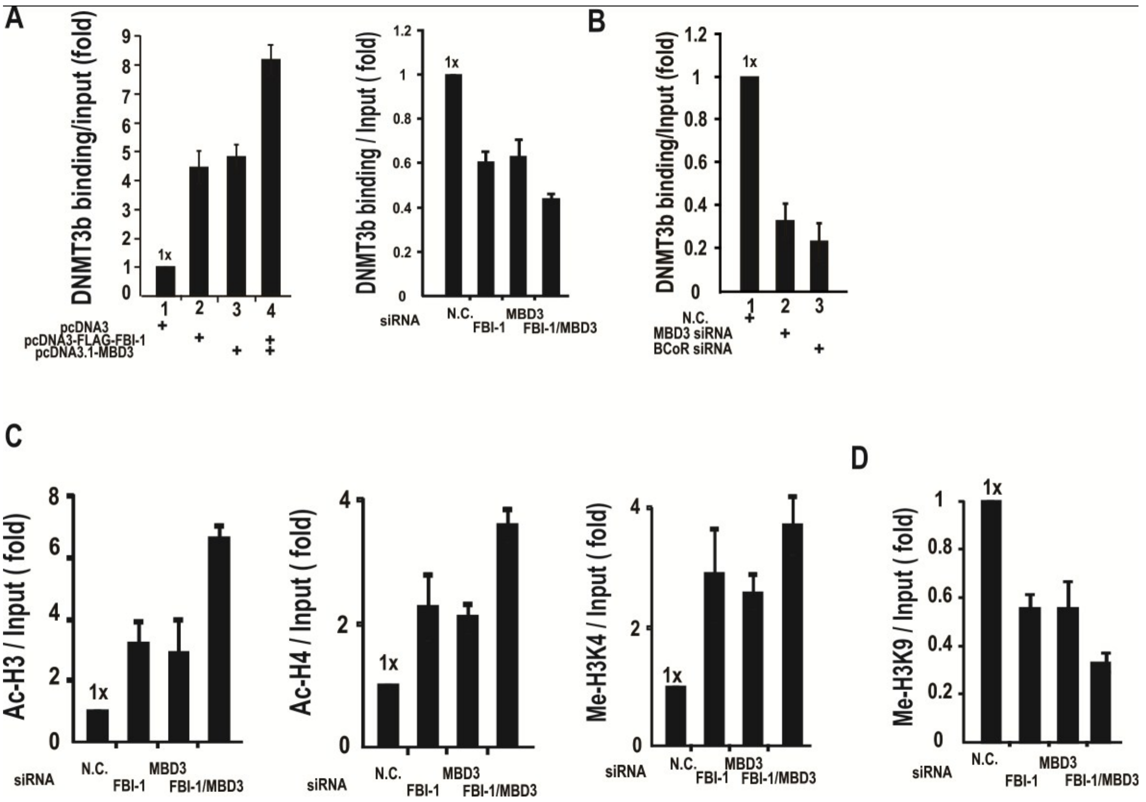

Supplementary Figure S4. ***Choi et al., 2012***

Supplementary Figure S5. Related to Figure 8. qChIP assays at the proximal promoter of *p21WAF/CDKN1A*. HP1 binding is affected by MBD3, FBI-1, but HP1 does not affect binding of FBI-1 and MBD3. HP1 weakly affects the binding of DNMTs, but significantly affects epigenetic histone marks. (A) qChIP assays of HP1 binding to the endogenous *CDKN1A* proximal promoter after knockdown of FBI-1 and/or MBD3. (B) qChIP assays. Knock-down of HP1 does not affect binding of FBI-1, MBD3, Mi-2/NuRD-HDAC complex. (C) qChIP assays. Knock-down of HP1 only weakly affects DNMTs binding. (D) qChIP assays. Knock-down of HP1 increases acetylated histones H3/H4 and H3K4-Me3, but decreases H3K9-Me3, a histone marker of transcription repression.

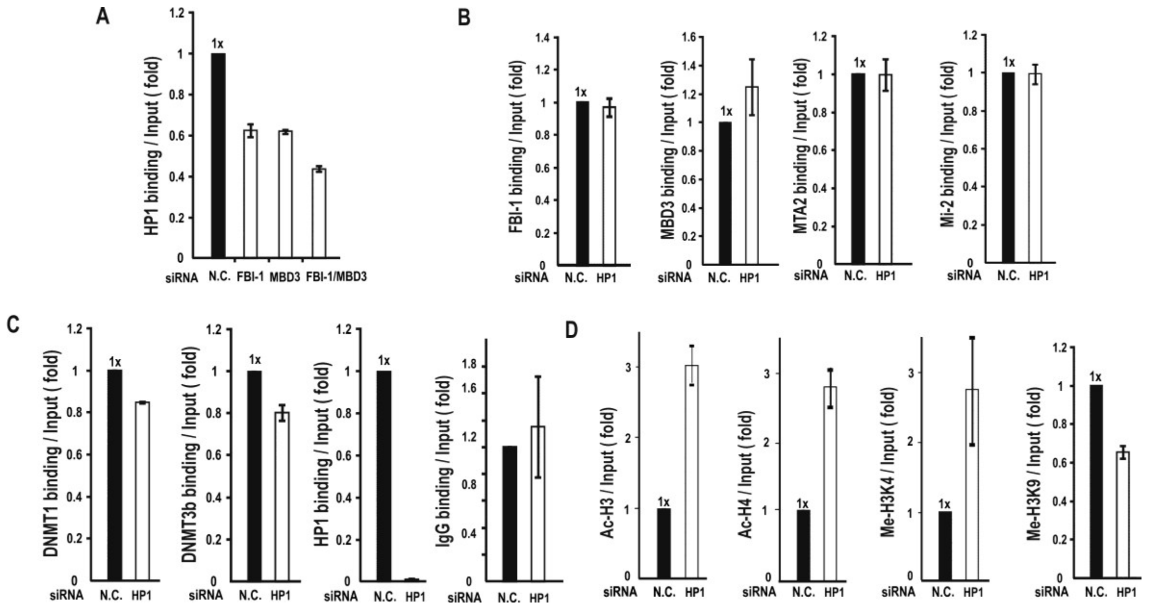

Supplementary Figure S5. ***Choi et al., 2012***

Supplementary Figure S6. Related to Figure 6A. TSA and 5-aza-2'-dC treatment do not affect the expression of endogenous FBI-1 and/or MBD3 expression at mRNA and protein levels. (A, B) Western blot and RT-qPCR assays of endogenous FBI-1 and MBD3 expression in the HEK293 cells treated with TSA (200 nM) or 5-aza-2'-dC (2 uM).

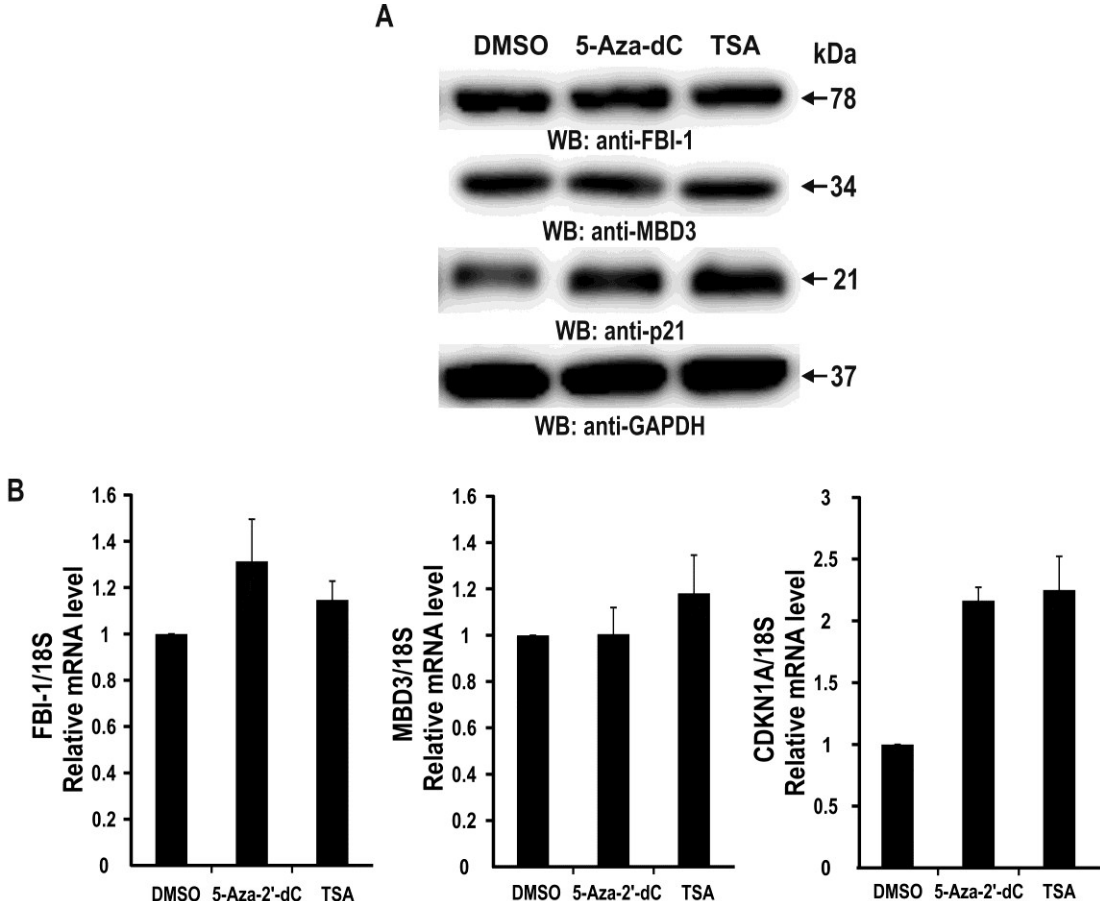

Supplementary Figure S6. ***Choi et al., 2012***

Supplementary Figure S7. Related to Figure 6(G, H). The proximal *CDKN1A* promoter can be repress by DNA methylation; FBI-1 and MBD3 are important in DNA methylation. (A, B) Bisulfite DNA sequencing assays. Shown is the map of methylated cytosines in the CDKN1A promoter. Cells were transfected with expression vectors or siRNA knocking down FBI-1 and/or MBD3, and DNA was prepared for bisulfite sequencing. Individual PCR products were cloned, and 20 clones were sequenced. Filled circles indicate methylated CpG sites; empty circle indicate unmethylated CpG sites.

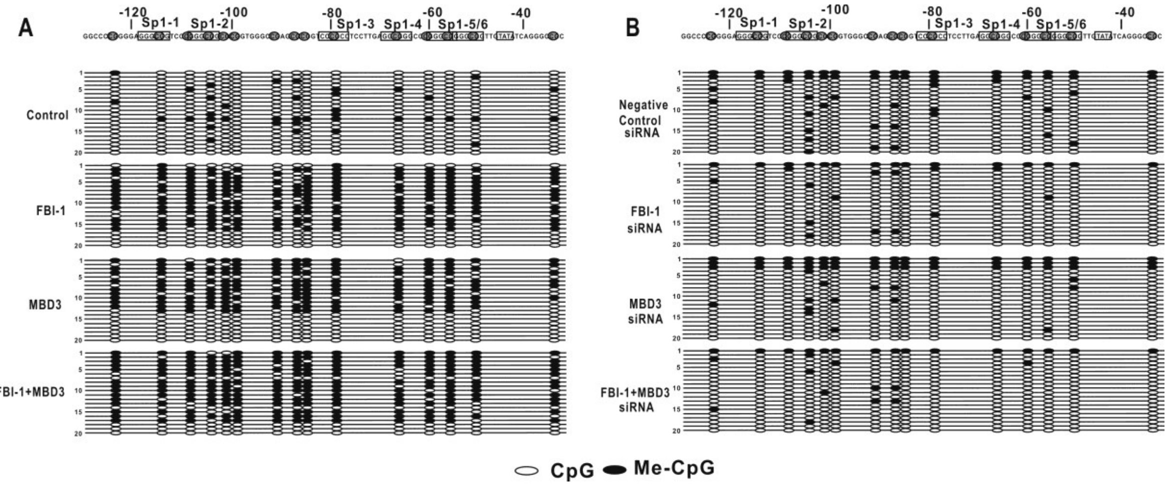

Supplementary Figure S7. ***Choi et al., 2012***

Supplementary Figure S8. Related to Figure 6E. FBI-1 overexpression combined with knock-down of MBD3 significantly affects the methylation of proximal *CDKN1A* promoter. (A) Me-DIP assays of DNA methylation at the proximal *CDKN1A* promoter in HEK293 cells with ectopic FBI-1 expression and/or knocked-down MBD3. Me-PC, methylation positive control; Me-NC, methylation negative control; GAPDH, GAPDH promoter; Alpha X1, X-linked alpha-satellites. (B) RT-qPCR analysis of the expression of FBI-1, MBD3, and p21 mRNA in HEK293 cells transfected with ectopic FBI-1 expression and/or knocked-down MBD3. Ectopic FBI-1 and/or MBD3 decrease endogenous p21 expression.

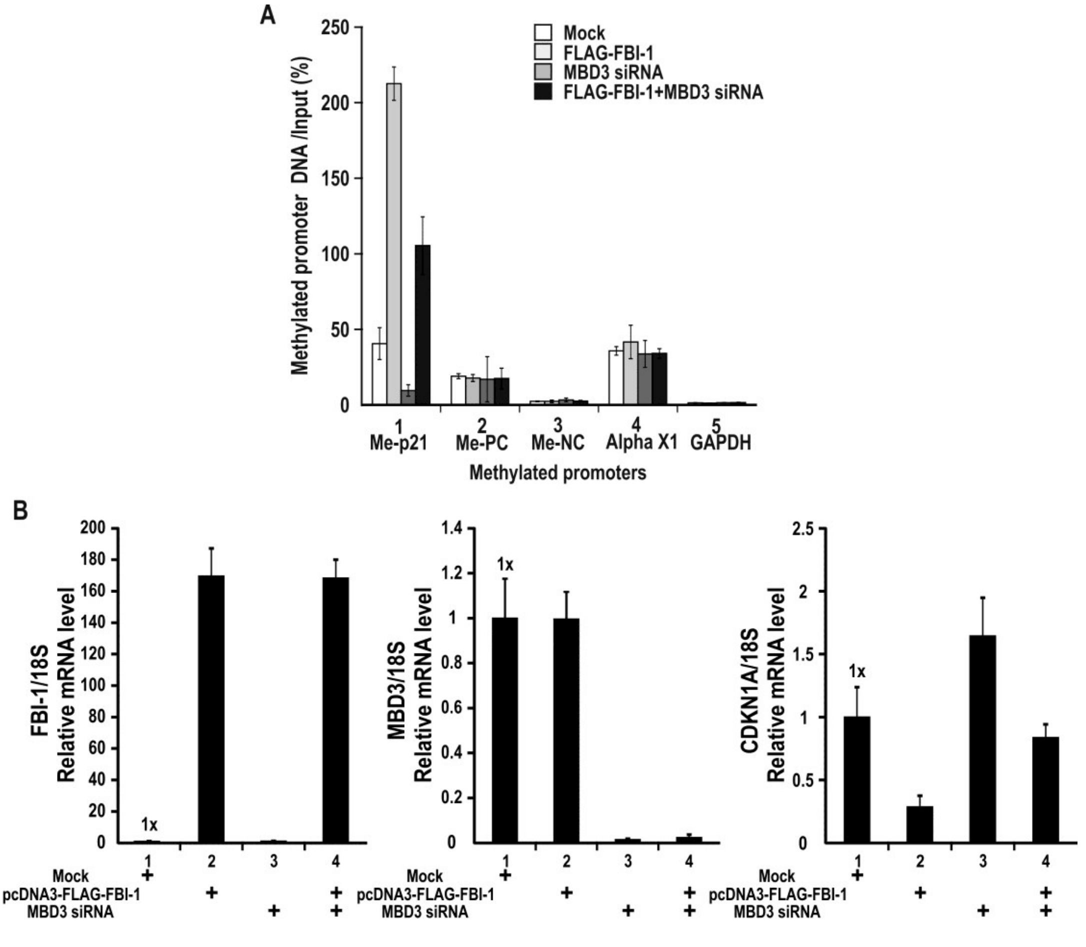

Supplementary Figure S8. ***Choi et al., 2012***

Supplementary Figure S9. Related to Figure 5I. BCoR does not affect interaction between FBI-1 and MBD3. Co-immunoprecipitation and western blot analysis of FBI-1, MBD3, and BCoR. HEK293 cells were transfected with BCoR siRNA and immunoprecipitated using an anti-FBI-1 antibody. The immunoprecipitates were analyzed for the presence of FBI-1, MBD3, and BCoR. GAPDH, Loading control.

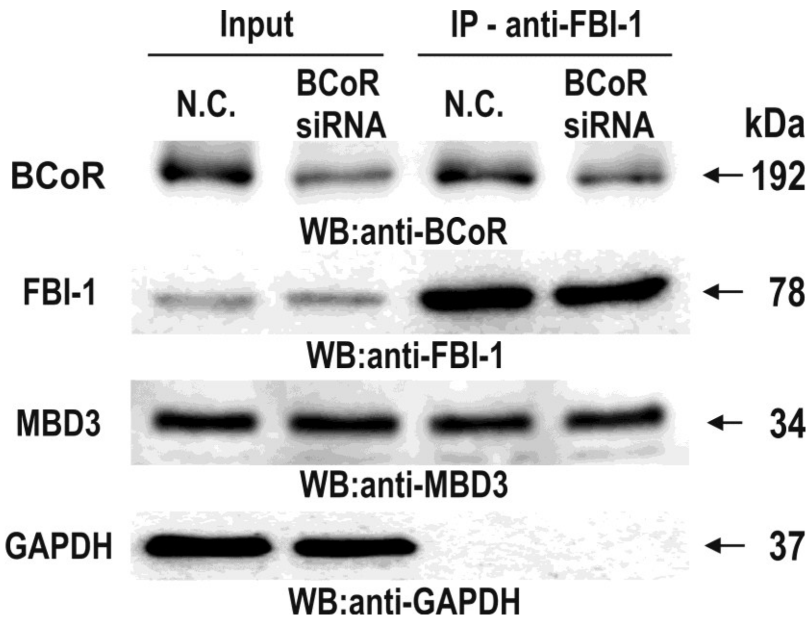

Supplementary Figure S9. ***Choi et al., 2012***
